# Designing Metabolic Division of Labor in Microbial Communities

**DOI:** 10.1128/mSystems.00263-18

**Published:** 2019-04-09

**Authors:** Meghan Thommes, Taiyao Wang, Qi Zhao, Ioannis C. Paschalidis, Daniel Segrè

**Affiliations:** a Department of Biomedical Engineering, Boston University, Boston, Massachusetts, USA; b Biological Design Center, Boston University, Boston, Massachusetts, USA; c Department of Electrical and Computer Engineering, Boston University, Boston, Massachusetts, USA; d Division of Systems Engineering, Boston University, Boston, Massachusetts, USA; e Department of Biology, Boston University, Boston, Massachusetts, USA; f Department of Physics, Boston University, Boston, Massachusetts, USA; g Bioinformatics Program, Boston University, Boston, Massachusetts, USA; University of California, San Diego

**Keywords:** division of labor, flux balance analysis, genome-scale stoichiometric models, metabolic networks, microbiome, mixed-integer linear programming, resource allocation, synthetic ecology, microbial communities

## Abstract

Microbes face a trade-off between being metabolically independent and relying on neighboring organisms for the supply of some essential metabolites. This balance of conflicting strategies affects microbial community structure and dynamics, with important implications for microbiome research and synthetic ecology. A “gedanken” (thought) experiment to investigate this trade-off would involve monitoring the rise of mutual dependence as the number of metabolic reactions allowed in an organism is increasingly constrained. The expectation is that below a certain number of reactions, no individual organism would be able to grow in isolation and cross-feeding partnerships and division of labor would emerge. We implemented this idealized experiment using *in silico* genome-scale models. In particular, we used mixed-integer linear programming to identify trade-off solutions in communities of Escherichia coli strains. The strategies that we found revealed a large space of opportunities in nuanced and nonintuitive metabolic division of labor, including, for example, splitting the tricarboxylic acid (TCA) cycle into two separate halves. The systematic computation of possible solutions in division of labor for 1-, 2-, and 3-strain consortia resulted in a rich and complex landscape. This landscape displayed a nonlinear boundary, indicating that the loss of an intracellular reaction was not necessarily compensated for by a single imported metabolite. Different regions in this landscape were associated with specific solutions and patterns of exchanged metabolites. Our approach also predicts the existence of regions in this landscape where independent bacteria are viable but are outcompeted by cross-feeding pairs, providing a possible incentive for the rise of division of labor.

**IMPORTANCE** Understanding how microbes assemble into communities is a fundamental open issue in biology, relevant to human health, metabolic engineering, and environmental sustainability. A possible mechanism for interactions of microbes is through cross-feeding, i.e., the exchange of small molecules. These metabolic exchanges may allow different microbes to specialize in distinct tasks and evolve division of labor. To systematically explore the space of possible strategies for division of labor, we applied advanced optimization algorithms to computational models of cellular metabolism. Specifically, we searched for communities able to survive under constraints (such as a limited number of reactions) that would not be sustainable by individual species. We found that predicted consortia partition metabolic pathways in ways that would be difficult to identify manually, possibly providing a competitive advantage over individual organisms. In addition to helping understand diversity in natural microbial communities, our approach could assist in the design of synthetic consortia.

## Abstract (precis)

Understanding how microbes assemble into communities is a fundamental open issue in biology, relevant to human health, metabolic engineering, and environmental sustainability. A possible mechanism for interactions of microbes is through cross-feeding, i.e., the exchange of small molecules. These metabolic exchanges may allow different microbes to specialize in distinct tasks and evolve division of labor. To systematically explore the space of possible strategies for division of labor, we applied advanced optimization algorithms to computational models of cellular metabolism. Specifically, we searched for communities able to survive under constraints (such as a limited number of reactions) that would not be sustainable by individual species. We found that predicted consortia partition metabolic pathways in ways that would be difficult to identify manually, possibly providing a competitive advantage over individual organisms. In addition to helping understand diversity in natural microbial communities, our approach could assist in the design of synthetic consortia.

## INTRODUCTION

Each microbial cell harbors a finite number of metabolic gene functions. The specific assortment of functions in a given organism thus represents the outcome of a trade-off between the cost of expressing different genes and the benefit of expression of those genes under conditions of different environments. This trade-off is considered to be one of the possible drivers of diversity in natural microbial communities, giving rise to metabolically differentiated groups rather than an individual superorganism (1–8). The emergence of metabolically differentiated subpopulations from isogenic populations has also been documented to occur in a fixed environment (9–21). The viability of coexisting populations of metabolically differentiated strains or species is often enabled by the exchange of metabolites (7, 11–13, 17, 18, 22–29). For example, initially identical populations of Escherichia coli that evolved on minimal glucose medium have been observed to give rise to a specialized subpopulation of cells that use the acetate secreted as a by-product of glucose fermentation (12, 13, 17). More broadly, metabolic interactions mediated by the exchange of small molecules help maintain the diversity and stability of natural microbial communities and allow communities to accomplish tasks that are metabolically intensive (5, 6, 23, 30). Moreover, obligate metabolic interdependencies (such as mutualism) are believed to contribute to the high prevalence of unculturability and fastidiousness among natural microbial strains (7, 31–35).

A recent and increasingly common strategy to study microbial interdependencies is the construction (or evolution) of artificial microbial consortia specifically designed to display obligate mutualism. Current approaches to building synthetic communities of interacting microbes have so far mainly relied on intuition about simple genetic perturbations that would cause organisms to engage in obligate cross-feeding. In these interactions, one strain is unable to synthesize an essential metabolite (e.g., an amino acid) that is supplied via overproduction or leakage by another strain (36–43). This ensures that the two strains require each other’s presence in order to grow. While interesting and valuable, these strategies explore only a small portion of the very large and complex space of possible environmental and organismal modifications; in principle, organisms may have the potential to display complex cross-feeding strategies for multiple metabolites simultaneously or in an environment-dependent manner (44, 45). In fact, given the complexity of metabolism and its evolutionary history, it is possible that naturally evolved cross-feeding strategies may involve complex metabolic mutualism beyond single amino acid exchanges (46). In particular, loss of functions in one organism due to compensation by others has been hypothesized to be widespread (47) and may involve multiple genes and complex pathway architectures (29). In the engineering of consortia for specific metabolic engineering tasks, exploring this larger space of possibilities may open up novel strategies for bioproduction.

Surveying the landscape of possible paths for metabolic differentiation leading to obligate mutualism is a combinatorially difficult problem. While future elaborations of existing methods for high-throughput genetic modifications (e.g., multiplex automated genome engineering [MAGE] [14]) may enable a systematic exploration of this space *in vivo*, computational models can provide a preliminary assessment of the landscape of possible strategies and of how these strategies depend on different constraints on metabolic network complexity. Constraint-based models of metabolic networks, such as flux balance analysis (FBA) (48–58), can be leveraged specifically to ask questions that cannot be easily addressed experimentally. FBA represents metabolism as a set of biochemical reactions inferred from genome annotations and literature curation and evaluates cellular metabolism as a resource allocation problem. Given a set of biochemical, thermodynamic, and environmental constraints, FBA uses linear programming to determine the distribution of fluxes through a reaction network that satisfies a given optimization objective. Typically, this objective is that of maximizing the flux through a biomass-producing reaction, so FBA determines how a cell should optimally allocate nutrients based on its environment and biochemical capabilities such that the growth rate is maximized. FBA has also been increasingly used to study metabolic interactions in microbial consortia (7, 8, 23, 27–29, 38, 49–56, 59–63), as well as to predict optimal genetic knockouts for metabolite production (48).

Here, we explore how metabolic differentiation emerges from an isogenic population by using a newly developed constraint-based modeling approach which we name “division of labor in metabolic networks” (DOLMN). In particular, using DOLMN, we explore the space of feasible single-strain or multistrain metabolic networks by systematically limiting the number of intracellular and transport reactions in each metabolic model. After introducing the mathematical and integer linear programming formulation of DOLMN, we illustrate its capabilities through an analysis of division of labor based on core carbon metabolism in E. coli (57). We next apply DOLMN to a genome-scale E. coli model (58) and show that metabolically differentiated and interdependent communities are able to exist under stricter reaction constraints than a single, isolated strain and are even able to outcompete the single strain in some cases. Our results broaden knowledge of the scope of possible metabolic interdependencies between metabolically different species, with applications in understanding diversity in natural microbial communities and in designing new artificial consortia.

## RESULTS

### A method to design division of labor in microbial communities.

**FIG 1:**
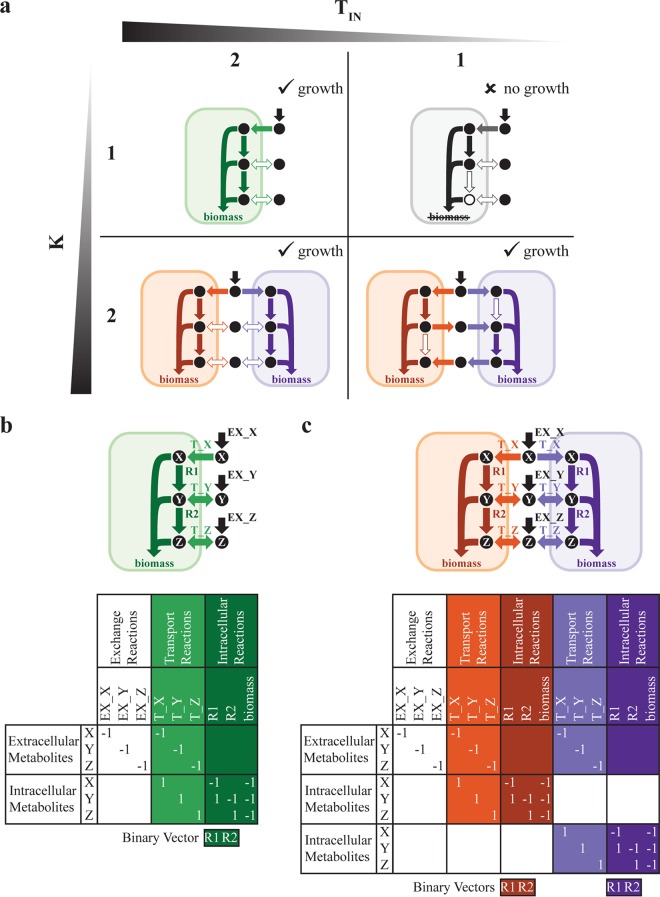
Division of labor in metabolic networks (DOLMN), illustrated as a toy model. (a) One- and two-strain solutions of a toy model. *K* indicates the number of target strains, and *T*_IN_ represents the constraint on the number of intracellular reactions allowed in each strain. Metabolites *X*, *Y*, and *Z* are required for each network to grow (i.e., produce biomass). (b and c) Two strains can exchange metabolites *Y* and *Z* (c), but a single strain can only take up environmental metabolites (b). (a) When *T*_IN_ = 2, 1- and 2-strain communities perform the same metabolic functions: uptake of metabolite *X*, conversion of metabolite *X* to metabolite *Y*, conversion of metabolite *Y* to metabolite *Z*, and creation of biomass from metabolites *X*, *Y*, and *Z*. The strains do not take up or secrete metabolite *Y* or metabolite *Z* (indicated as a hollow arrow). When *T*_IN_ = 1, the presence of an individual strain is no longer feasible because it cannot create metabolite *Z* (indicated as a hollow circle). The alternative solution, where reaction 1 is knocked out (not shown), is also infeasible because the single strain cannot then create metabolite *Y*. However, 2-strain communities are still feasible because the strains exchange metabolites *Y* and *Z*. If *T*_IN_ = 1 and the number of transport reactions allowed is constrained to two, then 2-strain communities are no longer feasible (not shown). (b and c) Toy metabolic network and corresponding (community) stoichiometric matrices and reaction binary vectors of 1-strain (b) and 2-strain (c) communities. The value of each stoichiometric coefficient represents the number of moles of each metabolite that participates in a reaction, with the sign indicating if a metabolite is a product (positive) or a reactant (negative). Exchange reactions are represented in black; transport reactions are represented in light green (b), orange (c), or purple (c); and intracellular reactions are represented in dark green (b), orange (c), or purple (c).

The problem of metabolic division of labor consists of partitioning a given metabolic network (which we refer to here as the “global network”) into subnetworks representing individual organisms (which we also call simply “strains” here) (see minimal example in Fig. 1). Each strain has its own metabolic network that includes intracellular reactions, as well as transport reactions, which determine how it interacts with the environment. Environmental availability of different nutrients is defined by constraints on the exchange reactions, which enable the inflow and outflow of environmental metabolites and by-products. In solving this problem concerning the division of labor, we make specific assumptions that reflect the nature and architecture of metabolic networks across different species as follows. (i) We do not set any specific *a priori* expectations about the presence of reactions in different strains. Consequently, a reaction from the global network may be selected to appear in one or more strains or may not appear in any strain at all. (ii) We expect each strain’s subnetwork to represent a well-connected, fully functional metabolism, so as to be capable of producing that strain’s biomass (see Materials and Methods). (iii) Upon simulation of cocultures of multiple coexisting strains, we require that all such strains must have equal growth rates, so that they would be able to stably coexist in a chemostat (8, 43, 64, 65).

To solve this problem, we devised division of labor in metabolic networks (DOLMN), which is formulated as a combinatorial optimization problem. Inputs of DOLMN consist of the global network (encoded in a stoichiometric matrix ***S*** and accompanied by upper and lower flux bounds, as in standard FBA formulations; see Materials and Methods); the number of target strains (*K*); and constraints on the number of intracellular (*T*_IN_) and transport (*T*_TR_) reactions allowed in each strain. Key outputs of DOLMN consist of a binary reaction vector (***t***) whose elements indicate whether a given reaction is present in a given strain and a continuous flux vector (***x***) for all reaction rates. Note that there is no specific requirement for two or more strains to end up using different reactions from the global network. A specific solution could entail multiple strains having exactly the same reactions and yet not interacting with each other. We expect division of labor to arise only upon making the *T*_IN_ or *T*_TR_ value too small for any individual strain to be able to survive without receiving specific molecular components from a metabolically distinct partner (Fig. 1a). Note that elements of ***t*** can switch on or off as a function of the current constraints, irrespective of their state under conditions of different constraints, and that *T*_TR_ does not differentiate between active transport and passive transport.

DOLMN, described in detail in Materials and Methods, involves the use of mixed integer linear programming (MILP). Our problem is NP (nondeterministic polynomial time) complete. It can be solved exactly for core metabolic network models (i.e., in a global network of ∼100 reactions), but it requires heuristics and long computational times for genome-scale models (∼1,000 reactions). Despite the use of the heuristic speed-up methods, we still obtain a single optimal solution for each value of *T*_TR_, *T*_IN_, and *K*.

### Metabolic division of labor in E. coli core metabolism.

**FIG 2:**
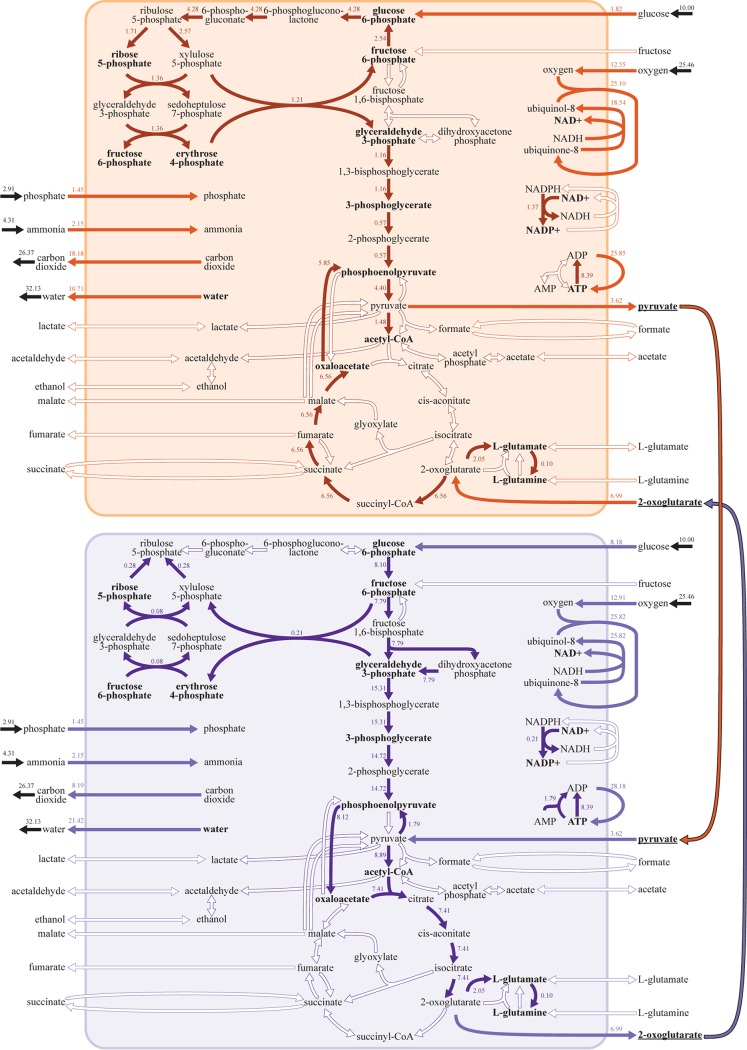
A DOLMN flux solution of E. coli core carbon metabolism. The figure illustrates the solution for 2-strain communities when 11 transport reactions (*T*_TR_ = 11) and 26 intracellular reactions (*T*_IN_ = 26) are allowed in the E. coli core network. Intracellular metabolites that are required for growth are bolded. Exchange reactions are represented in black and show the direction of the net flux between the two strains. Flux values (in millimoles per gram cell dry weight per hour) are indicated next to the arrows signifying the reactions. Both strains grew at 0.40 h^−1^. Transport reactions are represented in either light orange or purple and intracellular reactions are represented in either dark orange or purple for strain *x* or strain *y*, respectively. Reactions that the algorithm identifies as excluded or that have zero flux are indicated as a hollow arrow. The tricarboxylic acid (TCA) cycle is split between the two strains. Both strains consume oxygen, glucose, phosphate, and ammonium and secrete carbon dioxide and water. The two strains exchange the TCA intermediate 2-oxoglutarate and the glycolytic intermediate pyruvate (bolded and underlined); metabolite exchange is illustrated as an orange or purple arrow to indicate which strain is producing the metabolite.

As a first test and illustrative example of DOLMN, we investigated how E. coli core carbon metabolism (57) on minimal glucose medium would be partitioned between two strains (i.e., two trimmed versions of the E. coli core network) for a given limit on the number of allowed reactions (see Fig. 2 and Materials and Methods for details). Besides imposing constraints on the number of reactions allowed in each strain, we further required that both strains have the same growth rate of at least 0.1 h^−1^, effectively simulating stable coexistence in a chemostat (8, 43, 64, 65). Overall, we solved for 156 nontrivial combinations of *T*_TR_ and *T*_IN_, i.e., constraints under which either 1-strain or 2-strain communities could grow (65 nontrivial combinations for a single strain and 91 for two strains).

An interesting outcome of this analysis, obtained for subnetworks containing no more than 11 transport reactions and 26 intracellular reactions, was the discovery of a metabolic strategy in which each of two strains performs half of the tricarboxylic acid (TCA) cycle (Fig. 2). Both strains take up glucose, oxygen, phosphate, and ammonium; secrete carbon dioxide and water; perform glycolysis; and operate the nonoxidative phase of the pentose phosphate pathways (PPP). However, strain *x* (Fig. 2, orange) takes up four times as much glucose as strain *y* (Fig. 2, purple); operates the nonoxidative phase of the PPP in the forward direction; and utilizes different reactions in glycolysis. Furthermore, neither strain utilizes the glyoxylate shunt in the TCA cycle; instead, the metabolites oxaloacetate and 2-oxoglutarate split the TCA cycle into roughly two parts. Strain *y* performs the first part of the TCA cycle, converting oxaloacetate and acetyl-coenzyme A (acetyl-CoA) into 2-oxoglutarate and generating NADPH. Meanwhile, strain *x* generates oxaloacetate, NADH, and ATP through the transformation of 2-oxoglutarate in the second part of the TCA cycle.

Neither of the strains, in this case, was able to perform all needed metabolic functions without the inflow of specific metabolites produced by the partner. In particular, exchange of 2-oxoglutarate and pyruvate was necessary for survival of this 2-strain consortium (Fig. 2). Strain *x* performs a portion of the TCA cycle in order to produce 2-oxoglutarate, which is converted to biomass (as well as l-glutamate and l-glutamine, which are also converted to biomass). The excess 2-oxoglutarate is utilized by strain *y* for biomass as well as for the production of pyruvate. Pyruvate, in turn, is converted to biomass, and its excess is utilized by strain *x*.

This example illustrates how, even for a relatively small network, DOLMN can provide putative division of labor strategies that could not be easily designed manually. DOLMN could be similarly applied to other core metabolic models (66) such as have been generated for a large number of organisms.

### A growth landscape illustrates division of labor strategies in E. coli genome-scale metabolism.

We next applied DOLMN to a much larger global network, namely, genome-scale E. coli metabolism (58). In this case, individual strains found by the algorithm would represent E. coli variants with a reduced set of functionalities. Altogether, we solved for 5,347 combinations of *T*_TR_ and *T*_IN_ in which we found a feasible solution, including 738 for a single strain, 2,207 for two strains, and 2,402 for three strains.

**FIG 3:**
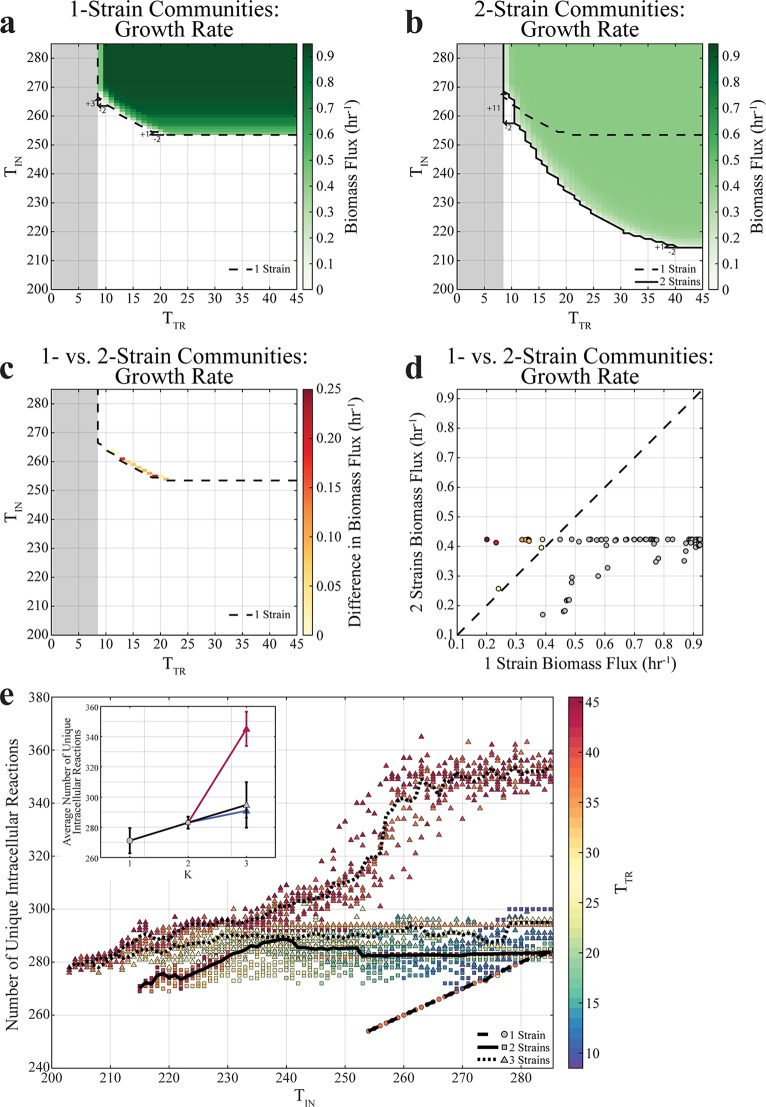
Growth (*T*_TR_,*T*_IN_) landscapes of 1- and 2-strain communities. The gray region depicts the infeasible region in which strains cannot grow because there are not enough transport reactions to take up and secrete metabolites. Strains were required to maintain a biomass flux of at least 0.1 h^−1^. Each data point represents a single simulation. (a) Growth landscape of 1-strain communities. Biomass flux values are interpolated to obtain values for the instances where the numbers of transport reactions (*T*_TR_) allowed are 23, 25 to 33, 35 to 37, and 39 to 45. In the instances where fewer transport constraints (decreasing *T*_TR_) are allowed, more intracellular reactions (*T*_IN_) are required. For example, when *T*_TR_ decreases from 21 to 19, only 1 additional intracellular reaction is required. However, when *T*_TR_ decreases from 11 to 9, 3 additional intracellular reactions are required. (b) Growth landscape of 2-strain communities. For example, when *T*_TR_ decreases from 41 to 39, *T*_TR_ only increases by 1, but when *T*_TR_ decreases from 11 to 9, *T*_TR_ increases by 11. (c) Growth landscape of the differences in growth rates between 2- and 1-strain communities, where the constraints under which 2-strain communities grow faster than 1-strain communities are indicated. Only those constraints under which 1- and 2-strain communities can both grow are included. (d) The growth rates of 2-strain communities compared to 1-strain communities. Gray circles represent constraints where 1-strain communities grow faster than 2-strain communities, and colored circles represent constraints where 2-strain communities grow faster than 1-strain communities. (e) The number of unique intracellular reactions in 1-, 2-, and 3-strain communities plotted as a function of *T*_IN_ and colored according to the value of *T*_TR_, where the number of unique intracellular reactions for multistrain communities represents the union of the intracellular reactions present in all strains. (Inset) Means and standard deviations of the number of unique intracellular reactions under all constraint conditions where 1-strain communities are viable. The blue and red lines indicate means and standard deviations of the two separate trajectories for 3-strain communities.

To display how growth rates vary as a function of *T*_TR_ and *T*_IN_, we systematically mapped the landscapes of possible 1-, 2-, and 3-strain simulations (Fig. 3a and b; see also Fig. S1a in the supplemental material). One first observation, consistent with expectations, was that as *T*_IN_ decreases (for an unconstrained number of transport reactions), individual strains reach a limit beyond which they cannot sustain growth, whereas consortia of two and three strains are still viable. In the example analyzed in Fig. 3, a 1-strain subnetwork needs at least 254 intracellular reactions to grow, whereas 2-strain subnetworks require only 215 intracellular reactions each and 3-strain subnetworks 203 intracellular reactions each (Fig. S2a). Interestingly, the 2-strain and 3-strain subnetworks have approximately the same number of intracellular reactions in common (Fig. S2b).

10.1128/mSystems.00263-18.1 FIG S1 (*T*_TR_,*T*_IN_) landscapes of 3-strain subnetworks. The gray region depicts the infeasible region in which strain subnetworks cannot grow because there are not enough transport reactions to take up and secrete metabolites. Strain subnetworks were required to maintain a biomass flux of at least 0.1 h^−1^. Each data point represents a single simulation. (a) Growth landscape of 3-strain subnetworks. (b and c) Growth landscape of the differences in growth rates between 3- and 1-strain subnetworks (b) and the differences in growth rates between 3- and 2-strain subnetworks (c), where the constraints under which 3-strain subnetworks grew faster than 1-strain (b) or 2-strain (c) subnetworks are indicated. Only constraints under which 1- and 3-strain subnetworks (b) or 2- and 3-strain subnetworks (c) can both grow are included. (d) Metabolic distance landscape of 3-strain subnetworks, averaged over the distances between strains *A* and *B*, *A* and *C*, and *B* and *C*. Strains that are more metabolically differentiated (have fewer reactions in common) are represented by a greater distance. (e and f) The growth rates of 3-strain communities compared to 1-strain (e) and 2-strain (f) communities. Gray circles represent constraints where 1-strain (e) or 2-strain (f) communities grew faster than 3-strain communities, and the colored circles represent constraints where 3-strain communities grew faster than 1-strain (e) or 2-strain (f) communities. (g) The number of metabolites exchanged between strains in 3-strain subnetwork simulations. Download FIG S1, TIF file, 1.8 MB. Copyright © 2019 Thommes et al. 2019 Thommes et al. This content is distributed under the terms of the Creative Commons Attribution 4.0 International license.

10.1128/mSystems.00263-18.2 FIG S2 (a) Minimum number of intracellular reactions needed for growth (*T*_IN_) as a function of the number of strains (*K*). (b) Number of intracellular reactions in common between 2 strains (orange) and 3 strains (purple) at their minimum *T*_IN_. (c) Transport reactions kept when the number of transport reactions allowed was 9. Download FIG S2, TIF file, 1.9 MB. Copyright © 2019 Thommes et al. 2019 Thommes et al. This content is distributed under the terms of the Creative Commons Attribution 4.0 International license.

**FIG 4:**
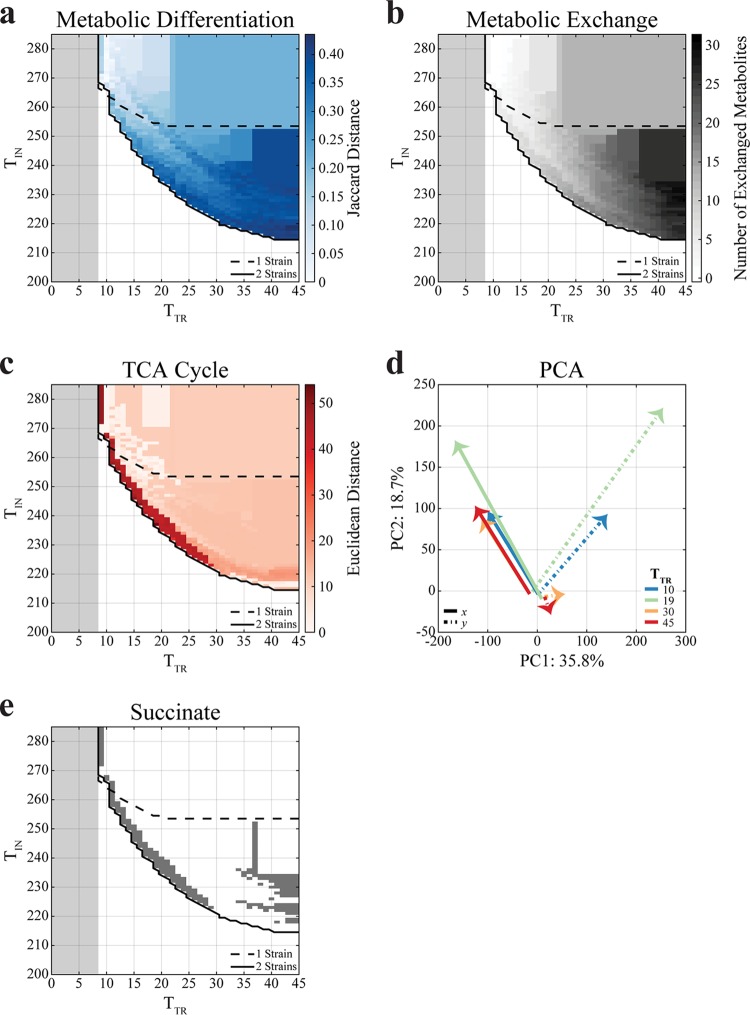
Landscapes (*T*_TR_,*T*_IN_) of metabolic differentiation and exchange in 2-strain communities. The gray region depicts the infeasible region in which strains cannot grow because there are not enough transport reactions for take-up and secretion of metabolites. Each data point represents a single simulation. (a) Jaccard distance between reaction binary vectors (***t***) in 2-strain community simulations. Strains that are more metabolically differentiated (have fewer reactions in common) have a greater distance. Two-strain communities are more metabolically differentiated when they are in the region where a single strain cannot grow. (b) The number of metabolites exchanged between strains in 2-strain community simulations. The number of exchanged metabolites increases as the constraint on the number of intracellular reactions becomes greater (decreasing *T*_IN_). As the constraint on the number of transport reactions becomes greater (decreasing *T*_TR_), the number of exchanged metabolites decreases. (c) Euclidean distance between the fluxes of TCA cycle reactions in 2-strain community simulations. (d) The principal-component analysis (PCA) plot of 2-strain communities at *T*_TR_ = 10, 19, 30, and 45, with arrows pointing from *T*_IN_ = 285 to the growth boundary (*T*_IN_ = 267, 237, 222, and 215, respectively). Solid lines denote strain *x* and dot-dash lines denote strain *y*. (e) Constraints under which the 2-strain communities exchange succinate. Two-strain communities distribute the TCA cycle under the same constraints as those under which they exchange succinate.

The observed landscapes display a fundamental nonlinear trade-off between minimizing *T*_IN_ (intracellular complexity) and minimizing *T*_TR_ (metabolic exchange). This nonlinearity implies that removing the same number of transport reactions at different points along the frontier of the feasible region can be compensated for by adding different numbers of intracellular reactions. For example, decreasing *T*_TR_ by 2 at large *T*_TR_ values can be compensated for by adding a single intracellular reaction (increasing *T*_IN_ by 1), while removing the same number of transport reactions at small *T*_TR_ would require a much larger compensation with intracellular reactions (Fig. 3a and b; see also Fig. S1a). It is important to note that decreasing the *T*_TR_ value negatively influences growth because it restricts not only each strain’s ability to take up metabolites but also its ability to secrete metabolites. If an organism cannot secrete metabolites, it accumulates waste (which results in an infeasible FBA solution). Irrespective of the number of strains in the community, it appears that the E. coli strain subnetworks require at least 9 transport reactions in order to support growth, corresponding to the main elemental sources and other irreducible biomass requirements; this strict bound is illustrated with a gray, shaded region in the (*T*_TR_,*T*_IN_) landscape (Fig. 3a to c; see also Fig. 4a, c, d, and e). Among other things, these transporters enable the strains to take up carbon, nitrogen, sulfate, and phosphate sources (Fig. S2c).

Further analysis of the landscapes for 1-, 2-, and 3-strain communities also reveals the existence of regions in which division of labor potentially provides a competitive advantage. Given that multiple strains coexisting in a consortium have to share available resources, they tend to grow more slowly than individually growing strains (Fig. 3a and b). One notable exception is a thin strip at the boundary, in which an individual strain can grow. At this frontier for a single strain, we observed that 2-strain communities can grow more rapidly than 1-strain communities (Fig. 3c and d). A biologically important implication of this result is the fact that the 2-strain communities would in principle have the chance to collectively outcompete the 1-strain ones. Similarly, 3-strain communities grow faster than 2-strain communities along the boundary in which 2-strain communities can grow (Fig. S1c and f) but only grow faster than 1-strain communities at a single constraint (Fig. S1b and e). These results suggest that the number of strains that achieve the highest growth rates under a given set of circumstances might naturally increase as environmental constraints tighten. This situation could arise, for example, if the burden of protein cost in the cell were to increase or if selection processes were to gradually come to favor streamlined strains (e.g., as previously observed experimentally and reported [10, 11, 14–21, 35]).

In examining the union of all intracellular reactions present at a particular constraint, we observed that the number of unique intracellular reactions seen with multistrain communities is larger than the number seen with a single strain and that 3-strain communities sometimes have higher numbers of unique intracellular reactions than 2-strain communities (Fig. 3e). The larger collective metabolic networks (corresponding to the more specialized subnetworks) likely facilitate growth under conditions of strict constraints, particularly below the boundary at which 1-strain communities can no longer exist, and are likely the result of metabolic division of labor. On the basis of these observations and on the results obtained for the core model (Fig. 2), we expect that the capacity of two or more E. coli strains to survive together under conditions of tighter constraints on the number of allowed reactions is due to metabolic division of labor and cross-feeding. We explore this in the next section.

### Emergence of obligate mutualism is coupled with sharp metabolic network differentiation and exchange of different chemicals.

A global overview of how metabolism enables the coexistence of 2- and 3-strain communities in the (*T*_TR_,*T*_IN_) landscape can be obtained by plotting the metabolic distance (see Materials and Methods) between the subnetworks of the strains (Fig. 4a; see also Fig. S1d) or the number of metabolites exchanged between them (Fig. 4b; see also Fig. S1g). For 2-strain communities, the fraction of reactions that the two strains have in common (i.e., the Jaccard similarity) tends to decrease overall as *T*_IN_ and *T*_TR_ decrease (Fig. 4a; see also Fig. S3a and b). This is due to the fact that, as the number of allowed reactions is reduced, the two strains can grow only if they take distinct metabolic roles (i.e., perform division of labor). Interestingly, despite the large metabolic network rearrangements induced by these constraints, the 2-strain consortia are able to maintain a fairly constant growth rate until they become infeasible (Fig. 3b). These division of labor strategies are also visible in terms of the number of metabolites that the 2-strain communities have to exchange with each other in order to grow (Fig. 4b; see also Fig. S3c).

10.1128/mSystems.00263-18.3 FIG S3 (a and b) Jaccard distance between 2-strain subnetwork reaction binary vectors (a) and the Euclidean distance between 2-strain subnetwork fluxes (b) as a function of *T*_IN_. The distance between 2-strain subnetworks generally decreases as *T*_IN_ increases, except when *T*_TR_ = 9 (a and b) and 11 (b). The number of exchanged metabolites increases as the Jaccard distance increases for 2-strain (c) and 3-strain (d) communities. (e) Phi coefficient of Jaccard distance or (f) point-biserial correlation of Euclidean distance and metabolite exchange profiles of 2-strain subnetwork simulations are indicated. Positive values indicate that a metabolite is exchanged when pathways are metabolically differentiated, and negative values indicate that a metabolite is not exchanged when pathways are metabolically differentiated. Download FIG S3, TIF file, 2.9 MB. Copyright © 2019 Thommes et al. 2019 Thommes et al. This content is distributed under the terms of the Creative Commons Attribution 4.0 International license.

**FIG 5:**
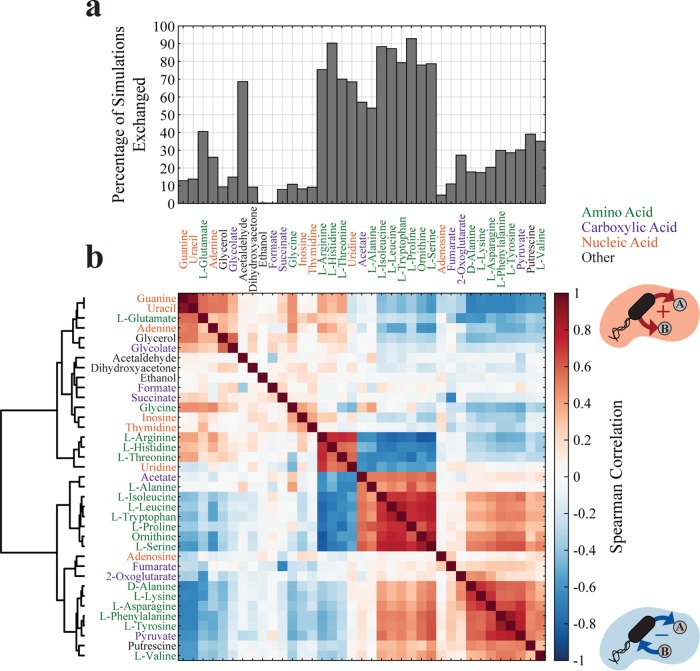
Characterization of exchanged metabolites for 2-strain community simulations. (a) Bar plot of the percentage of simulations in which a metabolite is exchanged in 2-strain communities, clustered hierarchically using Ward’s method. (b) Spearman correlation and associated dendrogram of exchanged metabolites, measuring the relationship of the metabolite exchange. A positive value specifies that both of the metabolites are secreted or taken up by a strain (only secretion is shown in the cartoon), and a negative value specifies that one metabolite is taken up and the other is secreted by a strain.

In short, multiple-strain communities can grow under stricter constraints on *T*_IN_ because they distribute metabolic reactions and exchange metabolites (Fig. S3c and d). Overall, different 2-strain communities can vary widely in terms of the metabolites being exchanged, with molecules ranging from central carbon compounds such as acetate and pyruvate to amino acids (Fig. 5a).

In order to gain better insight into the metabolic changes that accompany the rise in the number of pairs of obligate mutualistic strains, we reduced the multidimensional space of fluxes using principal-component analysis (PCA) (see Materials and Methods). Clusters in the principal-component space would indicate common metabolic strategies, hardly detectable through visual inspection of the network themselves; paths between these clusters would additionally portray how the strain subnetworks move through this metabolic space as constraints become more stringent. We observed three clusters, indicating three major metabolic strategies (Fig. 4d; see also Fig. S4). The largest cluster occurs at the origin and corresponds to large *T*_IN_ values. That is, for fairly unconstrained intracellular metabolism (i.e., when both strains are allowed a large number of intracellular reactions), all strains (for 1-strain and 2-strain simulations) use the same metabolic strategy for core energetic requirements. In particular, they display similar uses of the TCA cycle (Fig. 4c) and of other central carbon metabolism pathways (glycolysis/gluconeogenesis, oxidative phosphorylation; see Fig. S6). These metabolic regimes correspond to standard respiratory metabolism, in line with the expected metabolic fluxes for E. coli grown on minimal glucose medium (57, 58, 67). However, as *T*_IN_ becomes more constrained (i.e., as the number of intracellular reactions allowed decreases), the 2-strain subnetworks move away from each other, indicating that they diversify into different metabolic strategies. Note that the specific path that is followed by these networks as *T*_IN_ decreases is also a function of *T*_TR_ (Fig. 4d; see also Fig. S4).

10.1128/mSystems.00263-18.4 FIG S4 Principal-component analysis. Each subplot shows the PCA plot for strain *x* (solid line, downward triangle) and strain *y* (dotted line, upward triangle) in 2-strain subnetworks at the indicated *T*_TR_. The color of the marker indicates if the point represents a large *T*_IN_ (red) or small *T*_IN_ (purple). Download FIG S4, TIF file, 1.7 MB. Copyright © 2019 Thommes et al. 2019 Thommes et al. This content is distributed under the terms of the Creative Commons Attribution 4.0 International license.

To understand the different metabolic strategies associated with different clusters in the PCA, we calculated the Euclidean distance between pathways in each of the two strains in a 2-strain community and mapped it to the (*T_T_*_R_,*T*_IN_) landscape (see Materials and Methods). A striking outcome of this metabolic distance analysis was the fact that, for a broad range of *T*_TR_ values (e.g., values ranging from 9 to 29), the 2-strain subnetworks had a large difference in their TCA cycle (Fig. 4c), mirroring the observation reported for the core E. coli network (Fig. 2). This strategy of splitting the TCA cycle was also supplemented by large differences in glycolysis/gluconeogenesis, which were in fact observed along the entire *T*_IN_ boundary for 2-strain communities (Fig. S6), suggesting that this could represent a generalized version of the acetate utilization phenotype observed in classical evolutionary experiments (8, 12, 13). It is important to note that Jaccard distance values quantify differences in reaction content (Fig. S5), or in how the metabolic network was modified, whereas Euclidean distance values measure differences in reaction flux magnitude and direction (Fig. S6), or in how the modified network is being utilized.

10.1128/mSystems.00263-18.5 FIG S5 Jaccard (*T*_TR_ and *T*_IN_) landscapes of metabolic differentiation by pathway. Data represent the Jaccard distance between the reaction binary vectors (***t***) of each pathway in 2-strain subnetwork simulations. Strains that are more metabolically differentiated (have fewer reactions in common) have a greater distance. Download FIG S5, TIF file, 2.9 MB. Copyright © 2019 Thommes et al. 2019 Thommes et al. This content is distributed under the terms of the Creative Commons Attribution 4.0 International license.

10.1128/mSystems.00263-18.6 FIG S6 (*T*_TR_,*T*_IN_) Euclidean landscapes of metabolic differentiation by pathway. Euclidean distance between flux vectors (***x***) of each pathway in 2-strain subnetwork simulations. Strains that are more metabolically differentiated have a greater distance. Download FIG S6, TIF file, 2.9 MB. Copyright © 2019 Thommes et al. 2019 Thommes et al. This content is distributed under the terms of the Creative Commons Attribution 4.0 International license.

As indicated by the increase in the number of exchanged metabolites that occurs as constraints become tighter (Fig. 4b), the pairs of metabolically differentiated strains described above can survive due to metabolic cross-feeding. For example, in a 2-strain community, one of the strains could utilize the metabolites available from the environment and could secrete by-products that can enable the other strain to survive. We again used the (*T*_TR_,*T*_IN_) landscape to track how constraints affect the metabolites being exchanged (Fig. 4e; see also Fig. S7 and S8). Different metabolites display drastically different patterns (Fig. S7 and S8): some metabolites are exchanged almost universally (e.g., acetate) whereas others appear only in specific subregions of the landscape (e.g., l-glutamate below the *T*_IN_ boundary for 1-strain communities). Notably, succinate is shown to be exchanged predominantly at the *T*_IN_ boundary of 2-strain communities (Fig. 4e), in very close correspondence to the area of the landscape where the TCA cycle is drastically split (Fig. 4c). Furthermore, succinate exchange (Fig. 4e) and the metabolic differentiation of the TCA cycle (Fig. 4c) have a correlation coefficient of 0.63 (Fig. S3f). This suggests that, based on genome-scale simulations, succinate would be one of the key intermediates for the rise of E. coli strains surviving by using complementary halves of the TCA cycle.

10.1128/mSystems.00263-18.7 FIG S7 Metabolite exchange profiles for 2-strain subnetwork simulations. The constraints under which 2-strain subnetworks exchange metabolites are indicated. Download FIG S7, TIF file, 2.9 MB. Copyright © 2019 Thommes et al. 2019 Thommes et al. This content is distributed under the terms of the Creative Commons Attribution 4.0 International license.

10.1128/mSystems.00263-18.8 FIG S8 Metabolite exchange profiles for 3-strain subnetwork simulations. The constraints under which 3-strain subnetworks exchange metabolites are indicated. Download FIG S8, TIF file, 2.8 MB. Copyright © 2019 Thommes et al. 2019 Thommes et al. This content is distributed under the terms of the Creative Commons Attribution 4.0 International license.

### Fluxes of metabolic sharing can be strongly correlated.

Consortia of obligate symbiotic partners are predicted to emerge in regions of the (*T*_TR_,*T*_IN_) landscape where 1-strain communities are infeasible. As shown above, what makes these consortia viable (i.e., what makes it feasible for the corresponding strains to produce biomass despite the strong restriction on intracellular reactions) is the possibility of metabolite exchange between the 2-strain communities.

We thus sought to perform analyses of the metabolites being exchanged across the whole (*T*_TR_,*T*_IN_) landscape. In the specific setup used for the *in silico* experiments, among a total of 143 extracellular metabolites, only 37 (25.9%) metabolites were exchanged in at least one simulation of 2-strain communities. Most (78.4%) of these exchanged metabolites were exchanged in at least 10% of all coculture simulations (Fig. 5a). One class of abundantly exchanged molecules is the set of amino acids. These solutions can be viewed as similar to the artificially imposed auxotrophies used to engineer synthetic consortia (39–43, 68). Other frequently exchanged molecules include carboxylic acids (e.g., acetate and pyruvate) and nucleic acids (e.g., thymidine and adenine), which are known to be exchanged in natural communities (12, 13, 23, 61).

Additional insight can be gained by exploring the relationship between exchanged metabolites, i.e., by asking whether we should expect specific pairs of metabolites to be simultaneously exchanged in the same or opposite directions between two organisms. Knowledge of such correlations/anticorrelations may be useful as a strategy for choosing biomarkers (if two organisms exchange A, they are also likely to exchange B), as an indicator of fundamental metabolic trade-offs (X can provide A only if Y provides B), or as a broad suggestion of the existence of unavoidable couplings in the interactions present in a microbial community. Similar analyses of coupling between fluxes, e.g., through linear optimization (69–71), elementary flux modes (72), or Bayesian approaches (73), have become a broadly used way of determining structural network properties that can be helpful for metabolic engineering applications. Here, we sought to estimate correlations between exchange fluxes across an ensemble of networks with different stoichiometries [i.e., all the networks in the (*T*_TR_,*T*_IN_) landscape], making the use of existing algorithms challenging. We thus applied the Spearman correlation to exchange reaction fluxes (Fig. 5b) in order to measure if metabolites are exchanged jointly (both taken up or secreted by a strain [positive ρ]) or reciprocally (one is taken up and the other is secreted by a strain [negative ρ]). While Spearman correlation was chosen because we do not expect fluxes to scale linearly with *T*_TR_ and *T*_IN_ (Fig. S9c), we also computed Pearson correlations for the same data set (Fig. S9a), obtaining qualitatively similar results. Notably, the correlations described in detail below are substantially different from those that one would observe from applying a Bayesian flux coupling algorithm (73) to the original metabolic network for two coupled E. coli strains (Fig. S9b).

10.1128/mSystems.00263-18.9 FIG S9 (a) Pearson correlation and (b) Bayesian flux covariance of exchanged metabolites in 2-strain communities, clustered as described for Fig. 5b. (c to f) Scatter plot of exchange fluxes with noted Spearman and Pearson correlations. Download FIG S9, TIF file, 2.4 MB. Copyright © 2019 Thommes et al. 2019 Thommes et al. This content is distributed under the terms of the Creative Commons Attribution 4.0 International license.

Two major patterns emerged from this analysis. First, by looking at the hierarchically clustered correlation matrix, one can immediately recognize several block structures. The largest block structure seems dominated by amino acid exchange. In particular, two sets of (mostly) amino acids seem to be highly correlated within each set but to be highly anticorrelated across the sets. The first set includes l-arginine, l-histidine, l-threonine, and uridine, which are all correlated with each other, whereas the second set includes acetate, l-alanine, l-isoleucine, l-leucine, l-tryptophan, l-proline, ornithine, and l-serine. Interestingly, these anticorrelated block sets do not seem to map trivially to different precursor pathways (e.g., the upper versus lower glycolysis pathways), suggesting that other metabolic trade-offs may determine these patterns. One can also observe a second block structure dominated by amino acids that are all correlated, containing d-alanine, l-lysine, l-asparagine, l-phenylalanine, l-tyrosine, pyruvate, and—to a lesser extent—fumarate, 2-oxoglutarate, putrescine, and l-valine.

The second significant outcome of this correlation matrix is related to the TCA cycle-splitting result described above. In particular, succinate and fumarate are anticorrelated (ρ = −0.67), indicating reciprocal exchange. These two metabolites are intermediates of the TCA cycle and are involved in sequential steps, suggesting that this anticorrelation corresponds to the metabolic division of labor strategy characterized by splitting of the TCA cycle (Fig. 2). This is further confirmed by the fact that the region in the (*T*_TR_,*T*_IN_) landscape where succinate is exchanged matches very closely with the region in which the 2-strain communities show very distinct forms of use of the TCA cycle (Fig. 4c and e) and that the exchange profile of succinate is correlated to the metabolic differentiation of the TCA cycle (Fig. S3e and f).

## DISCUSSION

Although this study was purely computational, the data provide a new conceptual framework and new predictions, including specific testable modifications, and enable us to perform analyses that are currently beyond current experimental capabilities. The phenotypic space that we explore involves 5,000 *in silico* experiments on the full E. coli network, across 1-, 2-, and 3-strain communities, under conditions of multiple constraints on the number of transport and intracellular reactions. For each organism’s model, we take into account large numbers of variables (1,075 reactions and 761 metabolites in each strain). Efficient algorithms therefore represent a key step toward exploring possible strategies for division of labor in synthetic microbial communities and toward understanding how metabolic specialization may arise in natural microbial consortia.

The spontaneous emergence of division of labor in natural microbial systems is still a poorly understood process. One possible mechanism for this type of interdependence is the Black Queen hypothesis, on the basis of which division of labor could arise in complex microbial communities through the loss, instead of the gain, of functions that can be performed by “leaky” partners (29, 47). Our approach bears some analogies to the Black Queen hypothesis, in that it identifies network solutions that are reduced relative to those of an initial large network, generating networks of metabolite-mediated interdependencies. However, in our optimization algorithm, we assume that all strains are simultaneously constrained by the same maximal number of reactions allowed and thus that reductions in gene numbers occur in parallel in coexisting strains, generating complementary metabolic capabilities.

Our results may be relevant for microbial ecology of natural communities, as well as for the study of synthetic microbial consortia. Ongoing efforts in synthetic ecology are mostly focused on engineering metabolic dependencies by making one strain unable to produce a terminal biomass precursor (e.g., an amino acid) which is then provided by another strain. Although this strategy has yielded new insight into how microbes interact within communities, it may not reflect the possible complexity of natural metabolic interactions. Our approach has the capacity to uncover a much broader set of potential opportunities for obligate cross-feeding, including the exchange of metabolites that are part of core metabolic processes, or “deep symbiosis” between organisms.

The split TCA cycle that we present in Fig. 2 is an example of this potential deep symbiosis, which can be related to existing metabolic diversity across microbial taxa. The TCA cycle is traditionally described as a method to generate energy, but it is also essential for the production of essential precursor metabolites for cellular biosynthesis. The classic, cyclical form focuses on energy generation, but branched variants emphasize biosynthetic precursor production. These branched forms have been observed in bacteria and are the result of either an incomplete TCA cycle (66, 74–79) or differential regulation (76, 78, 80, 81). The enzyme 2-oxoglutarate dehydrogenase, which catalyzes the conversion of 2-oxoglutarate to succinyl-CoA, is often lacking in organisms with an incomplete TCA cycle (75–79). Furthermore, 2-oxoglutarate dehydrogenase is transcriptionally repressed in E. coli under anaerobic conditions (78, 80, 81) and has been found to show reduced metabolic flux in E. coli evolution experiments (82, 83). Due to the inability of these organisms to generate succinyl-CoA from 2-oxoglutarate, they instead reduce oxaloacetate to succinyl-CoA “in reverse.” Moreover, the phosphoenolpyruvate (PEP)-pyruvate-oxaloacetate node (also known as the anaplerotic node) is the metabolic link between the TCA cycle and glycolysis/gluconeogenesis and acts as a switch to control how carbon flux is distributed through central carbon metabolism (84).

Interestingly, the TCA cycle splitting that we observed in the model predictions occurred at the metabolites 2-oxoglutarate and oxaloacetate, both of which are used to create various amino acids. 2-Oxoglutarate is used to produce the amino acids glutamate, glutamine, proline, and arginine, whereas the amino acids aspartate, asparagine, methionine, threonine, isoleucine, and lysine can be produced from oxaloacetate. The biological significance of a split TCA cycle is also manifested in the patterns of secretion and uptake reported in the literature. In particular, both 2-oxoglutarate and pyruvate (the two exchanged metabolites) have known transporters in E. coli (85, 86). These metabolites are often observed in the extracellular environment (87–89), indicating that they are available for utilization. Moreover, cross-feeding of 2-oxoglutarate has been observed between Saccharomyces cerevisiae and lactic acid bacteria (24) and in “*Chlorochromatium aggregatum*” (25), a phototrophic consortium of the green sulfur bacterium *Chlorobium chlorochromatii* and a proteobacterium (*Comamonadaceae*). Consequently, there is evidence that some of the strategies representing the division of labor identified by our approach are similar to the pathway configurations of existing consortia.

Our predictions were initially produced under the assumption that the different perturbations applied by our method correspond to genetic modifications. However, they could equally be interpreted as being a consequence of instances of gene downregulation instead of gene loss (16). In other words, all the solutions found by DOLMN may in principle manifest themselves in the form of phenotypic differentiation within a population of cells. In a complex multicellular system (such as the human body), this could occur in the form of division of labor among cell types in different tissues, whereas in clonal populations of microbes, this could imply phenotypic variation due to heterogeneous gene expression (90–92). Single-cell studies of microbial physiology (16), aided by genome-scale models of metabolism, could help unravel both genomic and transcriptional variability potentially associated with division of labor in the microbial world.

Experimental testing of the proposed division of labor strategies in E. coli may prove challenging due to the multiplicity of gene deletions that would have to be simultaneously performed, although conceptually driven complex redesign of metabolic networks has been successfully implemented experimentally before (93, 94). Recently developed technologies, such as multiplex automated genome engineering (MAGE) (95), conjugative assembly genome engineering (CAGE) (96), and the clustered regularly interspaced short palindromic repeat (CRISPR)/Cas system (97), could in principle facilitate such an endeavor. Still, a challenge of implementing multiple targeted knockouts experimentally is the chance of encountering high-order epistatic interactions between genes that are difficult to predict computationally and that may result in nonviable strains. Thus, instead of implementing all of the genetic perturbations at once, it may be advisable to engineer increasingly complex interactions involving gradual modifications of different reactions and pathways. For example, rather than deleting genes, one could consider engineering promoters to reduce the flux through each reaction and potentially let the strains evolve in the laboratory. Alternatively, one could perform proteome perturbations through protein overexpression or energy dissipation (98).

Besides experimental testing, one could ask whether assortments of reactions similar to the ones predicted by DOLMN for our mutualistic E. coli strains can be found in existing organisms. For example, natural coexisting microbes may have identified, through long-term adaptation, metabolic strategies close to those predicted by DOLMN. This issue could be addressed by comparing the presence/absence of different metabolic enzymes in our predicted mutualistic strains to enzyme presence/absence profiles across microbial genomes coexisting in specific environments (99). A broader, related issue is whether the overall assortment of enzymes across individual species in complex communities is predictable on the basis of fundamental principles. Through a computationally improved future version of DOLMN, one could ask whether an ecosystem-level metabolic network could be correctly partitioned into metabolic networks that are representative of major observed taxa.

### Conclusion.

We computationally explored the possible ways in which sets of metabolic reactions can be distributed among interacting microbial strains, with the goal of better understanding the trade-off between metabolic self-reliance and mutualistic exchange. The mathematical problem of designing metabolically viable organisms and communities from a global set of possible reactions is a very difficult one. The approach, heuristics, and examples illustrated in this work show, however, that solutions identified by our algorithm are feasible and biologically interpretable. Although direct experimental testing of our predictions would require further preliminary computational work (e.g., complementing the current analysis with dynamic FBA and kinetic modeling) and laborious generation of modified strains, it is important that instances of division of labor similar to what we predicted may be found from further scrutiny of known symbiotic relationships (100, 101) and experimental evolution data (102). Thus, in addition to illustrating putative nontrivial avenues for engineering communities of codependent strains (synthetic ecology design), our approach could be viewed as a step toward addressing a broader, overarching issue, namely, whether fundamental design principles can help improve understanding of microbial ecosystem diversity and the metabolic network structure of individual organisms in natural communities. Specifically, one could imagine that abundant horizontal gene transfer and long-term selection processes in ecosystems may have acted over geological time to efficiently allocate genes into mutually dependent organisms, very much in the manner in which our algorithm does. With increasing computational power and further optimized algorithms, it may become possible to extend our approach to a larger number of organisms, with the potential of providing a general theoretical scaffold for understanding how environments shape division of labor strategies and microbial diversity.

## MATERIALS AND METHODS

### Flux balance analysis (FBA).

To mathematically formulate FBA, let **S** denote the stoichiometric matrix of dimensions *m* × *n*, where *m* is the number of metabolites and *n* is the number of metabolic fluxes. Metabolic fluxes are defined as vector ***x***, where ***x***_lb_ and ***x***_ub_ are the lower and upper bounds, respectively, of the metabolic fluxes. These bounds are implied by empirical evidence of irreversibility or by nutrient availability in the growth medium. The cellular objective is expressed as a vector of weight coefficients for each reaction (e.g., biomass), denoted by ***c***, and the optimal objective value is a scalar value corresponding to *Z*_opt_. The FBA problem is formulated as follows:

$$Z_{\text{opt}}\;={\text{ max}}_{x}\;c^{\prime }x$$

(1) such that Sx = 0

$$x_{\text{lb}}\leq x\leq x_{\text{ub}}$$

where **0** is the vector of all zeroes and the prime indicates transposition.

### Community-level flux balance analysis.

In order to perform FBA capturing all reactions spanning an entire microbial community, we introduce a “universal stoichiometric matrix,” also denoted by ***S***, which expresses the stoichiometric coefficients of all metabolic reactions in the community irrespective of the organism they belong to, as previously described (63). Specifically, $S\;\in \;{\mathbb{R}}^{M\times N}$, where *M* = *M*_e_ + *M*_i_ represents the number of distinct metabolites and *N* = *N*_e_ + *N*_t_ + *N*_i_ represents the number of distinct reactions (see Fig. S10a in the supplemental material). The distinct *M* metabolites consist of two types: *M*_e_ extracellular metabolites and *M*_i_ intracellular metabolites. There are 3 different types of reactions: *N_e_* extracellular reactions, *N*_t_ transport reactions, and *N*_i_ intracellular reactions. The availability of nutrients (extracellular metabolites) from the environment is encoded in the extracellular reactions, and intracellular reactions encode each organism’s metabolism. Organisms use transport reactions to move metabolites between their intracellular compartment, which is unique to each organism in the community, and the extracellular environment, which is shared by all organisms in the community.

10.1128/mSystems.00263-18.10FIG S10The structure of the stoichiometric matrices. (a) Structure of universal stoichiometric matrix **S**. Block **S**^e^ represents the set of extracellular reactions used to take up nutrients from or secrete nutrients into the environment. Blocks **S***^t^*^1^ and **S***^t^*^2^ represent the set of transport reactions between extracellular and intracellular metabolites. Block **S**^i^ represents the set of intracellular reactions among intracellular metabolites. (b) The structure of the stoichiometric matrix for the whole-community ${\text{S}}^{\text{c}}\in \;{\mathbb{R}}^{M_{c}\times N_{c}}$, with corresponding binary vector **t**. Download FIG S10, TIF file, 0.8 MB.Copyright © 2019 Thommes et al.2019Thommes et al.This content is distributed under the terms of the Creative Commons Attribution 4.0 International license.

### A method for metabolic division of labor.

We first reformulate the universal stoichiometric matrix ***S*** to construct putative stoichiometric matrices for each species in the community. In particular, we construct a community stoichiometric matrix ***S***^c^, whose structure is shown in Fig. S10b. The block matrices ***S***^e^, ***S***^t1^, ***S***^t2^, and ***S***^i^ in ***S***^c^ are consistent with those in ***S***. Organisms in the community share the same nutrients and extracellular reactions. Because there are *K* organisms in the community, we replicate the block [***S***^t2^, ***S***^i^] that includes transport reactions and intracellular reactions *K* times and diagonally arrange them in ***S***^c^. Similar compositions of stoichiometric matrices had used in previous work on community-level flux balance modeling (45, 103, 104).

After the intracellular metabolites are obtained via the transport reactions, intracellular reactions take place inside each organism. This construction leads to the community stoichiometric matrix $\;S^{\text{c}}\;\in \;{\mathbb{R}}^{M_{\text{c}}\times N_{\text{c}}}$, where *M*_c_ = *M*_e_ + *K* × *M*_i_ and *N*_c_ = *N*_e_ + *K* × (*N*_t_ + *N*_i_). Notice that ***S***_c_ has 1 block column for extracellular reactions (*N*_e_ columns) and *K* block columns (of dimension *N*_t_ + *N*_i_), one for each organism, including all transport and intracellular reactions.

To capture design choices, we introduce a binary putative vector ***t*** = (*t*_1_,…, *t*_N_*c*__), where $t_{j}\;\in \;\left\{0,1\right\}$ and *j* = 1,…, *N*_c_ is a binary variable, indicating whether the *j*-th reaction is included in the corresponding organism (Fig. S10b). With ***t*** and ***S***^c^ available, we can partition ***S***^c^ to *K* individual matrices, ***S***^k^, by removing column j with *t_j_* = 0.

The problem of identifying ***t*** can now be formulated as the following MILP problem:

$$\underset{\;x,t}{\max}c^{\prime }x$$

$$\text{s.t. }S^{\text{c}}x\;=\;0,$$

(2) $$x_{lb}\leq x\leq x_{\text{ub}}$$

$$\text{diag}\left(x_{\text{lb}}\right)t\leq \;x\;\leq \text{ diag}\left(x_{\text{ub}}\right)t,$$

$$t_{\text{i}}\in \left\{0,1\right\},$$

$$\;t_{\text{min}}\leq \mathrm{Rt}\leq t_{\text{max}},$$

where diag(**x**) denotes a diagonal matrix whose main diagonal consists of the elements of vector ***x***, ***R*** is a regularization matrix, and ***t***_min_ and ***t***_max_ are appropriately defined constant vectors. Specifically, by appropriately defining ***R***, ***t***_min_, and ***t***_max_, we can impose constraints on the number of internal and transport reactions for each organism.

### The first optimization problem.

Suppose there are *K* organisms. The upper bounds on the number of active transport reactions and intracellular reactions in each organism are *T*_TR_ and *T*_IN_, respectively. We let $x_{{\text{biom}}_{\text{k}}}$ denote the flux of the biomass reaction for each organism *k* = 1,…, *K*. We also let *TR_k_* and *IN_k_* denote the index sets of the transport reactions and intracellular reactions for each organism *k*, respectively. For example, suppose for organism *k* the transport reactions have indices 4 and 6 and the internal reactions have indices 2, 5, and 9. Then, *TR_k_* = {4,6} and ${\mathrm{IN}}_{k}\;=\;\{2,5,9\}$. Both of these sets are subsets of the entire reaction index set {1,…, *N*_c_} for the community. The MILP (as shown in problem 2) takes the following form:

$$\underset{\;x,t}{\max}c^{\prime }x$$

$$\text{s.t. }S^{\text{c}}x\;=\;0,$$

$$\;x_{\text{lb }}\leq \;x\;\leq \;x_{\text{ub}},$$

(3) $$\;x_{{\text{biom}}_{1}}\;=. . .=\;x_{{\text{biom}}_{K}}\;\geq 0.1,$$

$$\text{ diag}\left(x_{\text{lb}}\right)t\leq \;x\;\leq \text{ diag}\left(x_{\text{ub}}\right)t,$$

$$\;\underset{j\in TR_{k}}{\sum }t_{j}\leq T_{\mathrm{TR}},\;k\in \left\{1,\cdots K\right\},$$

$$\;\underset{\text{j}\in IN_{\text{k}}}{\sum }t_{j}\;\leq T_{\mathrm{IN}},\;k\in \left\{1,\cdots K\right\},$$

$$\;t_{j}\in \left\{0,1\right\}$$

Let x*,t* denote an optimal solution of the MILP problem above.

We note that solving such a large-scale MILP problem, which involves hundreds or thousands of integer variables, is computationally expensive. Our experiments suggest that solving problem 3 for a community of two E. coli core models can be done relatively quickly (on the order of minutes or hours). On the other hand, solving the problem for a community model of two iJR904 E. coli models is very time-consuming. We employ certain methods to speed up finding an optimal solution. One method leverages the fact that instances with similar values for *T*_TR_ and *T*_IN_ have similar sets of active reactions. Specifically, as we decrease the values of *T*_TR_ and *T*_IN_, we use the sets of active reactions corresponding to larger *T*_TR_ and *T*_IN_ values to generate feasible solutions that are offered as putative solutions to the MILP solver. This tends to drastically decrease solution times. A complementary approach uses a decomposition idea. In particular, for solving problems involving a community of *K* organisms, we can use solutions for *K* − 1 organisms and append solutions for the additional organism. This generates feasible solutions, and it is possible to search for an effective feasible solution by varying the way the *K*-organism community is decomposed into a (*K* − 1)-organism community and an additional organism.

### The second optimization problem.

In order to reduce levels of redundant fluxes in transport and intracellular reactions, a second optimization problem is introduced where the integer variables are fixed to the optimal solution t* of the first-stage problem (problem 3) and where the biomass fluxes are also set to the optimal values obtained by the first-stage problem (problem 3). Specifically, we use the following equations:

$$\underset{x}{\min}\;{\left\|\;x\;(N_{\text{e}}+1:\text{end})\right\|}_{1}$$

$$\text{s.t. }S^{\text{c}}x\;=\;0,$$

$$\;x_{\text{lb }}\leq \;x\;\leq \;x_{\text{ub}},$$

(4) $$\;x_{{\text{biom}}_{\text{k}}}\;=\;x_{{\text{biom}}_{k\prime }}^{*}\;k\in \left\{1,\cdots K\right\},$$

$$\text{ diag}\left(x_{\text{lb}}\right)t^{*}\leq \;x\;\leq \text{ diag}\left(x_{\text{ub}}\right)t^{*}$$

This problem minimizes the ℓ_1_ norm of the flux vector (excluding exchange reactions) to induce sparsity and can be rewritten as a linear programming problem.

### Data structure and analysis.

For each constraint on the number of transport reactions, *T*_TR_, DOLMN outputs a structure, *C* (“model”), for each strain. This structure *C* contains fields to indicate the constraint on the number of intracellular reactions, *T*_IN_ (“model.sparse_con”); the reaction names in the global network (“model.rxns”); the growth rates at each *T*_IN_ (“model.biomass”); the reaction flux values at each *T*_IN_ (“model.flux”); and the reaction binary integer values at each *T*_IN_ (“model.int”). For each value of *T*_IN_, *T*_TR_, and *K*, only a single simulation was performed.

The analysis of the DOLMN output is performed in several MATLAB scripts. All analyses are split between the core and full iJR904 E. coli model. The scripts dolmn1a_parse_iJR904.m and dolmn1b_parse_core.m apply the function *algorithm2models* to all of the DOLMN outputs for the full and core iJR904 E. coli models, respectively. The function *algorithm2models* parses *C* into individual metabolic models, calculates the exchange flux of each individual strain (instead of the community exchange flux), and identifies exchanged metabolites. The scripts dolmn2a_summary_iJR904.m and dolmn2b_summary_core.m restructure the data for plotting, calculate the Jaccard distance and exchange flux correlations, and perform standard PCA (MATLAB function *pca*). Interpolations for the constraint landscapes are performed in dolmn2a_summaryInterp_iJR904.m and dolmn2b_summaryInterp_core.m. The only exception is the flux coupling analysis, which is performed separately. All main text figures were plotted using dolmn3_figures_main.m, and all supplementary figures were plotted using dolmn4_figures_supp.m.

All data (raw and analyzed), functions, and scripts can be found in the GitHub repository (https://github.com/segrelab/dolmn). Analysis was performed with MATLAB 2017b. MILP problems were solved by the use of Gurobi 7.0 (105).

### E. coli models.

E. coli core (57) and genome-scale iJR904 (58) models were retrieved from the BiGG database (106). The models were downloaded as .mat files in the COBRA (COnstraints-Based Reconstruction and Analysis) format (107). Stoichiometric matrices were reformatted as a community stoichiometric matrix ***S***^c^, as previously described, and the results are shown in Fig. S10b. Reaction and metabolite names were reordered to correspond with the community stoichiometric matrix.

### Calculating net metabolite exchange flux from transport reaction flux.

Many metabolites have multiple transport reactions, which means that the net flux of a metabolite into or out of an organism (and thereby into or out of the environment) is typically represented by exchange reactions. However, in community flux balance analysis, organisms share exchange reactions (Fig. S10b). In order to determine if a strain subnetwork took up or secreted a metabolite in multistrain communities, we had to calculate the net metabolite exchange flux within each strain subnetwork. The calculated net metabolite exchange flux is the sum of all transport reactions (determined as follows):

(5) $$x_{e}^{k}\;=\;S^{t1,k}x_{t}^{k}\;$$

where $x_{e}^{k}$ is the exchange flux of organism *k*, $x_{t}^{k}$ is the transport flux of organism *k*, and ***S***^(^*^t^*^1,^*^k^*^)^ is the portion of the stoichiometric matrix for extracellular metabolites and transport reactions (see Fig. S10).

### Calculating metabolic differentiation.

Two metrics were used to measure metabolic differentiation based on intracellular reactions. Jaccard distance measures differences in reaction content and quantifies diversification at the level of network topology. In contrast, Euclidean distance evaluates differences in the magnitude and direction of reaction fluxes and therefore illustrates how the network topology is being utilized differently between strains. Both Jaccard and Euclidean distance metrics were used because, at the extreme, strains could have kept the same reactions (could have a zero Jaccard distance) but could have used the reactions differently (could have a nonzero Euclidean distance), as shown in Fig. S3a and b. The built-in MATLAB function *pdist2* was used to calculate Jaccard and Euclidean distances with the “jaccard” and “euclidean” metrics, respectively.

### Identifying exchanged metabolites.

Metabolites are exchanged if one strain secretes the metabolite (has a positive exchange flux) and the other takes it up (has a negative exchange flux). Metabolites that were part of the medium (e.g., hydrogen ions) were not considered to be exchanged even if one strain secreted the metabolite and the other took it up. We created the MATLAB function *identifyExchangedMets* to determine which metabolites, if any, are exchanged between strains.

### Principal-component analysis.

Principal-component analysis (PCA) was performed on the intracellular flux values of 1- and 2-strains using the built-in MATLAB function *pca*. The biomass flux was excluded from the intracellular reactions and was instead used to normalize the intracellular flux values.

### Correlation and hierarchical clustering of exchange reaction flux.

The MATLAB function *corr* was used to calculate the Spearman and Pearson correlations of exchange reaction fluxes, normalized by biomass flux, providing information on how metabolites are exchanged together. The Spearman correlation of exchange reaction fluxes was hierarchically clustered by the inner squared Euclidean distance (Ward’s method) using the MATLAB functions *linkage* and *dendrogram*.

### Flux coupling analysis.

Flux coupling analysis was performed using the Bayesian metabolic flux analysis (73) MATLAB function *bfba*, which evaluates reaction fluxes as distributions instead of a single value. We sampled FBA solutions with 200 samples of 5 independent Markov chain Monte Carlo methods for a total of 1,000 flux vector samples, which represent all possible flux configurations compatible under the given constraints of the use of minimal glucose medium. We used thinning and accepted only every 1,000th flux sample to reduce the correlation of successive Markov chain Monte Carlo samples. Solutions were obtained with Gurobi (105) as the solver, the Gibbs sampler, and a 0.01 steady-state error value on mass balance. Flux coupling was indicated with flux sampling covariances, as in the function *plotfluxcov*.

### Data availability.

All data generated and analyzed in this study and the corresponding codes are available in the GitHub repository (https://github.com/segrelab/dolmn).

## References

[1] Lindemann SR, Bernstein HC, Song H-S, Fredrickson JK, Fields MW, Shou W, Johnson DR, Beliaev AS 2016 Engineering microbial consortia for controllable outputs. ISME J 10:2077–2084. doi:10.1038/ismej.2016.26. 26967105 PMC4989317

[2] D’Souza G, Waschina S, Pande S, Bohl K, Kaleta C, Kost C 2014 Less is more: selective advantages can explain the prevalent loss of biosynthetic genes in bacteria. Evolution 68:2559–2570. doi:10.1111/evo.12468. 24910088

[3] McCutcheon JP, Moran NA 2011 Extreme genome reduction in symbiotic bacteria. Nat Rev Microbiol 10:13–26. doi:10.1038/nrmicro2670. 22064560

[4] Zhou K, Qiao K, Edgar S, Stephanopoulos G 2015 Distributing a metabolic pathway among a microbial consortium enhances production of natural products. Nat Biotechnol 33:377–383. doi:10.1038/nbt.3095. 25558867 PMC4867547

[5] Klitgord N, Segrè D 2010 The importance of compartmentalization in metabolic flux models: yeast as an ecosystem of organelles, p 41–55. *In* Genome Informatics 2009. Proceedings of the 9th Annual International Workshop on Bioinformatics and Systems Biology (IBSB 2009). Imperial College Press, London, United Kingdom. 20238418

[6] Klitgord N, Segrè D 2011 Ecosystems biology of microbial metabolism. Curr Opin Biotechnol 22:541–546. doi:10.1016/j.copbio.2011.04.018. 21592777

[7] Zelezniak A, Andrejev S, Ponomarova O, Mende DR, Bork P, Patil KR 2015 Metabolic dependencies drive species co-occurrence in diverse microbial communities. Proc Natl Acad Sci U S A 112:6449–6454. doi:10.1073/pnas.1421834112. 25941371 PMC4443341

[8] Carlson RP, Beck AE, Phalak P, Fields MW, Gedeon T, Hanley L, Harcombe WR, Henson MA, Heys JJ 2018 Competitive resource allocation to metabolic pathways contributes to overflow metabolisms and emergent properties in cross-feeding microbial consortia. Biochm Soc Trans 46:269–284. doi:10.1042/BST20170242. 29472366

[9] Sandberg TE, Lloyd CJ, Palsson BØ, Feist AM 2017 Laboratory evolution to alternating substrate environments yields distinct phenotypic and genetic adaptive strategies. Appl Environ Microbiol 83:e00410-17. doi:10.1128/AEM.00410-17. 28455337 PMC5478979

[10] Friesen ML, Saxer G, Travisano M, Doebeli M 2004 Experimental evidence for sympatric ecological diversification due to frequency-dependent competition in Escherichia coli. Evolution 58:245–260. doi:10.1554/03-369. 15068343

[11] Elena SF, Lenski RE 2003 Evolution experiments with microorganisms: the dynamics and genetic bases of adaptation. Nat Rev Genet 4:457–469. doi:10.1038/nrg1088. 12776215

[12] Treves DS, Manning S, Adams J 1998 Repeated evolution of an acetate-crossfeeding polymorphism in long-term populations of Escherichia coli. Mol Biol Evol 15:789–797. doi:10.1093/oxfordjournals.molbev.a025984. 9656481

[13] Rosenzweig RF, Sharp RR, Treves DS, Adams J 1994 Microbial evolution in a simple unstructured environment: genetic differentiation in Escherichia coli. Genetics 137:903–917. 7982572 10.1093/genetics/137.4.903 PMC1206068

[14] van Gestel J, Vlamakis H, Kolter R 2015 Division of labor in biofilms: the ecology of cell differentiation. Microbiol Spectr 3:MB-0002-2014. doi:10.1128/microbiolspec.MB-0002-2014. 26104716

[15] Le Gac M, Brazas MD, Bertrand M, Tyerman JG, Spencer CC, Hancock REW, Doebeli M 2008 Metabolic changes associated with adaptive diversification in Escherichia coli. Genetics 178:1049–1060. doi:10.1534/genetics.107.082040. 18245349 PMC2248342

[16] Rosenthal AZ, Qi Y, Hormoz S, Park J, Li S-J, Elowitz MB 2018 Metabolic interactions between dynamic bacterial subpopulations. Elife 7:e33099. doi:10.7554/eLife.33099. 29809139 PMC6025961

[17] Rozen DE, Lenski RE 2000 Long-term experimental evolution in Escherichia coli. VIII. Dynamics of a balanced polymorphism. Am Nat 155:24–35. doi:10.1086/303299. 10657174

[18] Rozen DE, Schneider D, Lenski RE 2005 Long-term experimental evolution in Escherichia coli. XIII. Phylogenetic history of a balanced polymorphism. J Mol Evol 61:171–180. doi:10.1007/s00239-004-0322-2. 15999245

[19] Spencer CC, Bertrand M, Travisano M, Doebeli M 2007 Adaptive diversification in genes that regulate resource use in Escherichia coli. PLoS Genet 3:0083–0088. 10.1371/journal.pgen.0030015 PMC1779306 17238290

[20] Spencer CC, Tyerman J, Bertrand M, Doebeli M 2008 Adaptation increases the likelihood of diversification in an experimental bacterial lineage. Proc Natl Acad Sci U S A 105:1585–1589. doi:10.1073/pnas.0708504105. 18216261 PMC2234188

[21] van Gestel J, Vlamakis H, Kolter R 2015 From cell differentiation to cell collectives: Bacillus subtilis uses division of labor to migrate. PLoS Biol 13:e1002141. doi:10.1371/journal.pbio.1002141. 25894589 PMC4403855

[22] Zomorrodi AR, Segrè D 2016 Synthetic ecology of microbes: mathematical models and applications. J Mol Biol 428:837–861. doi:10.1016/j.jmb.2015.10.019. 26522937 PMC4798899

[23] Embree M, Liu JK, Al-Bassam MM, Zengler K 2015 Networks of energetic and metabolic interactions define dynamics in microbial communities. Proc Natl Acad Sci U S A 112:15450–15455. doi:10.1073/pnas.1506034112. 26621749 PMC4687543

[24] Ponomarova O, Gabrielli N, Sévin DC, Mülleder M, Zirngibl K, Bulyha K, Andrejev S, Kafkia E, Typas A, Sauer U, Ralser M, Patil KR 2017 Yeast creates a niche for symbiotic lactic acid bacteria through nitrogen overflow. Cell Syst 5:345–357.e6. doi:10.1016/j.cels.2017.09.002. 28964698 PMC5660601

[25] Müller J, Overmann J 2011 Close interspecies interactions between prokaryotes from sulfureous environments. Front Microbiol 2:146. doi:10.3389/fmicb.2011.00146. 21779277 PMC3132602

[26] Kastman EK, Kamelamela N, Norville JW, Cosetta CM, Dutton RJ, Wolfe BE 2016 Biotic interactions shape the ecological distributions of Staphylococcus species. mBio 7:e01157-16. doi:10.1128/mBio.01157-16. 27795388 PMC5082897

[27] Harcombe WR, Riehl WJ, Dukovski I, Granger BR, Betts A, Lang AH, Bonilla G, Kar A, Leiby N, Mehta P, Marx CJ, Segrè D 2014 Metabolic resource allocation in individual microbes determines ecosystem interactions and spatial dynamics. Cell Rep 7:1104–1115. doi:10.1016/j.celrep.2014.03.070. 24794435 PMC4097880

[28] Ponomarova O, Patil KR 2015 Metabolic interactions in microbial communities: untangling the Gordian knot. Curr Opin Microbiol 27:37–44. doi:10.1016/j.mib.2015.06.014. 26207681

[29] Zomorrodi AR, Segrè D 2017 Genome-driven evolutionary game theory helps understand the rise of metabolic interdependencies in microbial communities. Nat Commun 8:1563. doi:10.1038/s41467-017-01407-5. 29146901 PMC5691134

[30] Tsoi R, Wu F, Zhang C, Bewick S, Karig D, You L 2018 Metabolic division of labor in microbial systems. Proc Natl Acad Sci U S A 115:2526–2531. doi:10.1073/pnas.1716888115. 29463749 PMC5877992

[31] Pande S, Kost C 2017 Bacterial unculturability and the formation of intercellular metabolic networks. Trends Microbiol 25:349–361. doi:10.1016/j.tim.2017.02.015. 28389039

[32] Epstein SS 2013 The phenomenon of microbial uncultivability. Curr Opin Microbiol 16:636–642. doi:10.1016/j.mib.2013.08.003. 24011825

[33] Bernstein D, Dewhirst F, Segre D 2018 Quantifying biosynthetic network robustness across the human oral microbiome. bioRxiv https://www.biorxiv.org/content/10.1101/392621v1.

[34] Pande S, Merker H, Bohl K, Reichelt M, Schuster S, De Figueiredo LF, Kaleta C, Kost C 2014 Fitness and stability of obligate cross-feeding interactions that emerge upon gene loss in bacteria. ISME J 8:953–962. doi:10.1038/ismej.2013.211. 24285359 PMC3996690

[35] Ferea TL, Botstein D, Brown PO, Rosenzweig RF 1999 Systematic changes in gene expression patterns following adaptive evolution in yeast. Proc Natl Acad Sci U S A 96:9721–9726. doi:10.1073/pnas.96.17.9721. 10449761 PMC22277

[36] Harcombe WR 2010 Novel cooperation experimentally evolved between species. Evolution 64:2166–2172. doi:10.1111/j.1558-5646.2010.00959.x. 20100214

[37] Bernstein HC, Paulson SD, Carlson RP 2012 Synthetic Escherichia coli consortia engineered for syntrophy demonstrate enhanced biomass productivity. J Biotechnol 157:159–166. doi:10.1016/j.jbiotec.2011.10.001. 22015987 PMC3549274

[38] Henry CS, Bernstein HC, Weisenhorn P, Taylor RC, Lee JY, Zucker J, Song HS 2016 Microbial community metabolic modeling: a community data-driven network reconstruction. J Cell Physiol 231:2339–2345. doi:10.1002/jcp.25428. 27186840 PMC5132105

[39] Hoek TA, Axelrod K, Biancalani T, Yurtsev EA, Liu J, Gore J 24 8 2016 Resource availability modulates the cooperative and competitive nature of a microbial cross-feeding mutualism. PLoS Biol doi:10.1371/journal.pbio.1002540. PMC4996419 27557335

[40] Kerner A, Park J, Williams A, Lin XN 2012 A programmable Escherichia coli consortium via tunable symbiosis. PLoS One 7:e34032. doi:10.1371/journal.pone.0034032. 22479509 PMC3316586

[41] Wintermute EH, Silver PA 2010 Emergent cooperation in microbial metabolism. Mol Syst Biol 6:407. doi:10.1038/msb.2010.66. 20823845 PMC2964121

[42] Mee MT, Collins JJ, Church GM, Wang HH 2014 Syntrophic exchange in synthetic microbial communities. Proc Natl Acad Sci U S A 111:E2149–E2156. doi:10.1073/pnas.1405641111. 24778240 PMC4034247

[43] Shou W, Ram S, Vilar J 2007 Synthetic cooperation in engineered yeast populations. Proc Natl Acad Sci U S A 104:1877–1882. doi:10.1073/pnas.0610575104. 17267602 PMC1794266

[44] Pacheco AR, Moel M, Segre D 2018 Costless metabolic secretions as drivers of interspecies interactions in microbial ecosystems. bioRxiv https://www.biorxiv.org/content/10.1101/300046v1. 10.1038/s41467-018-07946-9 PMC6327061 30626871

[45] Klitgord N, Segrè D 2010 Environments that induce synthetic microbial ecosystems. PLoS Comput Biol 6:e1001002. doi:10.1371/journal.pcbi.1001002. 21124952 PMC2987903

[46] Medlock GL, Carey MA, McDuffie DG, Mundy MB, Giallourou N, Swann JR, Kolling GL, Papin JA 2018 Inferring metabolic mechanisms of interaction within a defined gut microbiota. Cell Syst 7:245–257.e7. doi:10.1016/j.cels.2018.08.003. 30195437 PMC6166237

[47] Morris JJ, Lenski RE, Zinser ER 2012 The black queen hypothesis: evolution of dependencies through adaptive gene loss. mBio 3:e00036-12. doi:10.1128/mBio.00036-12. 22448042 PMC3315703

[48] Burgard AP, Pharkya P, Maranas CD 2003 OptKnock: a bilevel programming framework for identifying gene knockout strategies for microbial strain optimization. Biotechnol Bioeng 84:647–657. doi:10.1002/bit.10803. 14595777

[49] Bauer E, Thiele I 2018 From network analysis to functional metabolic modeling of the human gut microbiota. mSystems 3:e00209-17. doi:10.1128/mSystems.00209-17. 29600286 PMC5872302

[50] O’Brien EJ, Monk JM, Palsson BØ 2015 Using genome-scale models to predict biological capabilities. Cell 161:971–987. doi:10.1016/j.cell.2015.05.019. 26000478 PMC4451052

[51] Magnúsdóttir S, Heinken A, Kutt L, Ravcheev DA, Bauer E, Noronha A, Greenhalgh K, Jäger C, Baginska J, Wilmes P, Fleming RMT, Thiele I 2016 Generation of genome-scale metabolic reconstructions for 773 members of the human gut microbiota. Nat Biotechnol 35:81–89. doi:10.1038/nbt.3703. 27893703

[52] Biggs MB, Papin JA 2017 Managing uncertainty in metabolic network structure and improving predictions using EnsembleFBA. PLoS Comput Biol 13:e1005413. doi:10.1371/journal.pcbi.1005413. 28263984 PMC5358886

[53] Biggs MB, Medlock GL, Kolling GL, Papin JA 2015 Metabolic network modeling of microbial communities. Wiley Interdiscip Rev Syst Biol Med 7:317–334. doi:10.1002/wsbm.1308. 26109480 PMC4575871

[54] Kreimer A, Doron-Faigenboim A, Borenstein E, Freilich S 2012 NetCmpt: a network-based tool for calculating the metabolic competition between bacterial species. Bioinformatics 28:2195–2197. doi:10.1093/bioinformatics/bts323. 22668793

[55] Freilich S, Zarecki R, Eilam O, Segal ES, Henry CS, Kupiec M, Gophna U, Sharan R, Ruppin E 2011 Competitive and cooperative metabolic interactions in bacterial communities. Nat Commun 2:589. doi:10.1038/ncomms1597. 22158444

[56] Levy R, Carr R, Kreimer A, Freilich S, Borenstein E 2015 NetCooperate: a network-based tool for inferring host-microbe and microbe-microbe cooperation. BMC Bioinformatics 16:164. doi:10.1186/s12859-015-0588-y. 25980407 PMC4434858

[57] Orth JD, Fleming RMT, Palsson BØ 8 9 2009, posting date. Reconstruction and use of microbial metabolic networks: the core Escherichia coli metabolic model as an educational guide. EcoSal Plus 2009 doi:10.1128/ecosalplus.10.2.1. 26443778

[58] Reed JL, Vo TD, Schilling CH, Palsson BØ 2003 An expanded genome-scale model of Escherichia coli K-12 (iJR904 GSM/GPR). Genome Biol 4:R54. doi:10.1186/gb-2003-4-9-r54. 12952533 PMC193654

[59] Chiu H-C, Levy R, Borenstein E 2014 Emergent biosynthetic capacity in simple microbial communities. PLoS Comput Biol 10:e1003695. doi:10.1371/journal.pcbi.1003695. 24992662 PMC4084645

[60] Levy R, Borenstein E 2013 Metabolic modeling of species interaction in the human microbiome elucidates community-level assembly rules. Proc Natl Acad Sci U S A 110:12804–12809. doi:10.1073/pnas.1300926110. 23858463 PMC3732988

[61] McNally CP, Borenstein E 2018 Metabolic model-based analysis of the emergence of bacterial cross-feeding via extensive gene loss. BMC Syst Biol 12:69. doi:10.1186/s12918-018-0588-4. 29907104 PMC6003207

[62] Stolyar S, Van Dien S, Hillesland KL, Pinel N, Lie TJ, Leigh JA, Stahl DA 2007 Metabolic modeling of a mutualistic microbial community. Mol Syst Biol 3:92. doi:10.1038/msb4100131. 17353934 PMC1847946

[63] Khandelwal RA, Olivier BG, Röling WFM, Teusink B, Bruggeman FJ 2013 Community flux balance analysis for microbial consortia at balanced growth. PLoS One 8:e64567. doi:10.1371/journal.pone.0064567. 23741341 PMC3669319

[64] Toprak E, Veres A, Yildiz S, Pedraza JM, Chait R, Paulsson J, Kishony R 2013 Building a morbidostat: an automated continuous-culture device for studying bacterial drug resistance under dynamically sustained drug inhibition. Nat Protoc 8:555–567. doi:10.1038/nprot.nprot.2013.021. 23429717 PMC3708598

[65] Smith HL, Waltman P 1995 The theory of the chemostat: dynamics of microbial competition. Cambridge University Press, Cambridge, United Kingdom.

[66] Edirisinghe JN, Weisenhorn P, Conrad N, Xia F, Overbeek R, Stevens RL, Henry CS 2016 Modeling central metabolism and energy biosynthesis across microbial life. BMC Genomics 17:568. doi:10.1186/s12864-016-2887-8. 27502787 PMC4977884

[67] Caglar MU, Houser JR, Barnhart CS, Boutz DR, Carroll SM, Dasgupta A, Lenoir WF, Smith BL, Sridhara V, Sydykova DK, Vander Wood D, Marx CJ, Marcotte EM, Barrick JE, Wilke CO 18 4 2017 The E. coli molecular phenotype under different growth conditions. Sci Rep 7:45303. doi:10.1038/srep45303. 28417974 PMC5394689

[68] Gore J, Youk H, van Oudenaarden A 2009 Snowdrift game dynamics and facultative cheating in yeast. Nature 459:253–256. doi:10.1038/nature07921. 19349960 PMC2888597

[69] Burgard AP, Nikolaev EV, Schilling CH, Maranas CD 2004 Flux coupling analysis of genome-scale metabolic network reconstructions. Genome Res 14:301–312. doi:10.1101/gr.1926504. 14718379 PMC327106

[70] Tefagh M, Boyd SP 10 12 2018 Quantitative flux coupling analysis. J Math Biol doi:10.1007/s00285-018-1316-9. 30535964

[71] Larhlimi A, David L, Selbig J, Bockmayr A 22 4 2012 F2C2: a fast tool for the computation of flux coupling in genome-scale metabolic networks. BMC Bioinformatics doi:10.1186/1471-2105-13-57. PMC3515416 22524245

[72] Klamt S, Regensburger G, Gerstl MP, Jungreuthmayer C, Schuster S, Mahadevan R, Zanghellini J, Müller S 13 4 2017 From elementary flux modes to elementary flux vectors: metabolic pathway analysis with arbitrary linear flux constraints. PLoS Comput Biol doi:10.1371/journal.pcbi.1005409. PMC5390976 28406903

[73] Heinonen M, Osmala M, Mannerström H, Wallenius J, Kaski S, Rousu J, Lähdesmäki H 2018 Bayesian metabolic flux analysis reveals intracellular flux couplings. arXiv https://arxiv.org/abs/1804.06673. 10.1093/bioinformatics/btz315 PMC6612884 31510676

[74] Huynen MA, Dandekar T, Bork P 1999 Variation and evolution of the citric-acid cycle: a genomic perspective. Trends Microbiol 7:281–291. doi:10.1016/S0966-842X(99)01539-5. 10390638

[75] Heinken A, Khan MT, Paglia G, Rodionov DA, Harmsen HJM, Thiele I 2014 Functional metabolic map of Faecalibacterium prausnitzii, a beneficial human gut microbe. J Bacteriol 196:3289–3302. doi:10.1128/JB.01780-14. 25002542 PMC4135701

[76] Steinhauser D, Fernie AR, Araújo WL 2012 Unusual cyanobacterial TCA cycles: not broken just different. Trends Plant Sci 17:503–509. doi:10.1016/j.tplants.2012.05.005. 22658681

[77] Greenfield S, Claus GW 1972 Nonfunctional tricarboxylic acid cycle and the mechanism of glutamate biosynthesis in Acetobacter suboxydans. J Bacteriol 112:1295–1301. 4640504 10.1128/jb.112.3.1295-1301.1972 PMC251562

[78] Wood AP, Aurikko JP, Kelly DP 2004 A challenge for 21st century molecular biology and biochemistry: What are the causes of obligate autotrophy and methanotrophy? FEMS Microbiol Rev 28:335–352. doi:10.1016/j.femsre.2003.12.001. 15449607

[79] Pearce J, Leach CK, Carr NG 1969 The incomplete tricarboxylic acid cycle in the blue-green alga Anabaena variabilis. J Gen Microbiol 55:371–378. doi:10.1099/00221287-55-3-371. 5783887

[80] Guest JR 1992 Oxygen-regulated gene expression in Escherichia coli. J Gen Microbiol 138:2253–2263. doi:10.1099/00221287-138-11-2253. 1479352

[81] Cronan JE Jr, Laporte D 28 6 2006, posting date. Tricarboxylic acid cycle and glyoxylate bypass. EcoSal Plus 2006 doi:10.1128/ecosalplus.3.5.2. 26443520

[82] Long CP, Gonzalez JE, Feist AM, Palsson BØ, Antoniewicz MR 2018 Dissecting the genetic and metabolic mechanisms of adaptation to the knockout of a major metabolic enzyme in Escherichia coli. Proc Natl Acad Sci U S A 115:222–227. doi:10.1073/pnas.1716056115. 29255023 PMC5776819

[83] Long CP, Gonzalez JE, Feist AM, Palsson BØ, Antoniewicz MR 2017 Fast growth phenotype of E. coli K-12 from adaptive laboratory evolution does not require intracellular flux rewiring. Metab Eng 44:100–107. doi:10.1016/j.ymben.2017.09.012. 28951266 PMC5845443

[84] Sauer U, Eikmanns BJ 2005 The PEP-pyruvate-oxaloacetate node as the switch point for carbon flux distribution in bacteria. FEMS Microbiol Rev 29:765–794. doi:10.1016/j.femsre.2004.11.002. 16102602

[85] Bramley HF, Kornberg HL 1987 Sequence homologies between proteins of bacterial phosphoenolpyruvate-dependent sugar phosphotransferase systems: identification of possible phosphate-carrying histidine residues. Proc Natl Acad Sci U S A 84:4777–4780. doi:10.1073/pnas.84.14.4777. 3299373 PMC305188

[86] Seol W, Shatkin AJ 1992 Escherichia coli α-ketoglutarate permease is a constitutively expressed proton symporter. J Biol Chem 267:6409–6413. 1556144

[87] Taymaz-Nikerel H, de Mey M, Ras C, ten Pierick A, Seifar RM, van Dam JC, Heijnen JJ, van Gulik WM 2009 Development and application of a differential method for reliable metabolome analysis in Escherichia coli. Anal Biochem 386:9–19. doi:10.1016/j.ab.2008.11.018. 19084496

[88] Bolten CJ, Kiefer P, Letisse F, Portais JC, Wittmann C 2007 Sampling for metabolome analysis of microorganisms. Anal Chem 79:3843–3849. doi:10.1021/ac0623888. 17411014

[89] Paczia N, Nilgen A, Lehmann T, Gätgens J, Wiechert W, Noack S 11 9 2012 Extensive exometabolome analysis reveals extended overflow metabolism in various microorganisms. Microb Cell Fact 11:122. doi:10.1186/1475-2859-11-122. 22963408 PMC3526501

[90] Nikolic N, Barner T, Ackermann M 15 11 2013 Analysis of fluorescent reporters indicates heterogeneity in glucose uptake and utilization in clonal bacterial populations. BMC Microbiol 13:258. doi:10.1186/1471-2180-13-258. 24238347 PMC3840653

[91] Elowitz MB, Levine AJ, Siggia ED, Swain PS 2002 Stochastic gene expression in a single cell. Science 297:1183. doi:10.1126/science.1070919. 12183631

[92] Ackermann M 2015 A functional perspective on phenotypic heterogeneity in microorganisms. Nat Rev Microbiol 13:497–508. doi:10.1038/nrmicro3491. 26145732

[93] Bar-Even A, Noor E, Lewis NE, Milo R 2010 Design and analysis of synthetic carbon fixation pathways. Proc Natl Acad Sci U S A 107:8889–8894. doi:10.1073/pnas.0907176107. 20410460 PMC2889323

[94] Antonovsky N, Gleizer S, Noor E, Zohar Y, Herz E, Barenholz U, Zelcbuch L, Amram S, Wides A, Tepper N, Davidi D, Bar-On Y, Bareia T, Wernick DG, Shani I, Malitsky S, Jona G, Bar-Even A, Milo R 23 6 2016 Sugar synthesis from CO_2_ in Escherichia coli. Cell doi:10.1016/j.cell.2016.05.064. PMC4930481 27345370

[95] Wang HH, Isaacs FJ, Carr PA, Sun ZZ, Xu G, Forest CR, Church GM 2009 Programming cells by multiplex genome engineering and accelerated evolution. Nature 460:894–898. doi:10.1038/nature08187. 19633652 PMC4590770

[96] Ma NJ, Moonan DW, Isaacs FJ 2014 Precise manipulation of bacterial chromosomes by conjugative assembly genome engineering. Nat Protoc 9:2285–2300. doi:10.1038/nprot.2014.081. 25188631 PMC5568562

[97] Cong L, Ran FA, Cox D, Lin S, Barretto R, Habib N, Hsu PD, Wu X, Jiang W, Marraffini LA, Zhang F 2013 Multiplex genome engineering using CRISPR/Cas systems. Science 339:819–823. doi:10.1126/science.1231143. 23287718 PMC3795411

[98] Basan M, Hui S, Okano H, Zhang Z, Shen Y, Williamson JR, Hwa T 2015 Overflow metabolism in Escherichia coli results from efficient proteome allocation. Nature 528:99–104. doi:10.1038/nature15765. 26632588 PMC4843128

[99] Kanehisa M, Sato Y, Furumichi M, Morishima K, Tanabe M 2018 New approach for understanding genome variations in KEGG. Nucleic Acids Res 47:590–595. doi:10.1093/nar/gky962. PMC6324070 30321428

[100] Glaeser J, Overmann J 2004 Biogeography, evolution, and diversity of epibionts in phototrophic consortia. Appl Environ Microbiol 70:4821–4830. doi:10.1128/AEM.70.8.4821-4830.2004. 15294820 PMC492462

[101] Moran NA, McCutcheon JP, Nakabachi A 2008 Genomics and evolution of heritable bacterial symbionts. Annu Rev Genet 42:165–190. doi:10.1146/annurev.genet.41.110306.130119. 18983256

[102] Good BH, McDonald MJ, Barrick JE, Lenski RE, Desai MM 2017 The dynamics of molecular evolution over 60,000 generations. Nature 551:45–50. doi:10.1038/nature24287. 29045390 PMC5788700

[103] Zomorrodi AR, Maranas CD 2012 OptCom: a multi-level optimization framework for the metabolic modeling and analysis of microbial communities. PLoS Comput Biol 8:e1002363. doi:10.1371/journal.pcbi.1002363. 22319433 PMC3271020

[104] Zhao Q, Segrè D, Paschalidis IC 2016 Optimal allocation of metabolic functions among organisms in a microbial ecosystem, p 7063–7068. *In* 55th IEEE Conference on Decision and Control (CDC), Las Vegas, NV. IEEE, New York, NY.

[105] Gurobi. 2018 Gurobi Optimizer reference manual. Gurobi, Frankfurt am Main, Germany.

[106] King ZA, Lu J, Dräger A, Miller P, Federowicz S, Lerman JA, Ebrahim A, Palsson BØ, Lewis NE 2016 BiGG Models: a platform for integrating, standardizing and sharing genome-scale models. Nucleic Acids Res 44:D515–D522. doi:10.1093/nar/gkv1049. 26476456 PMC4702785

[107] Schellenberger J, Que R, Fleming RMT, Thiele I, Orth JD, Feist AM, Zielinski DC, Bordbar A, Lewis NE, Rahmanian S, Kang J, Hyduke DR, Palsson BØ 2011 Quantitative prediction of cellular metabolism with constraint-based models: the COBRA Toolbox v2.0. Nat Protoc 6:1290–1307. doi:10.1038/nprot.2011.308. 21886097 PMC3319681

