# Supplementary figures and images for: Designing Metabolic Division of Labor in Microbial Communities
Source: mSystems. 2019 Apr 9;4(2):e00263-18. doi: 10.1128/mSystems.00263-18 (PMC6456671; doi:10.1128/mSystems.00263-18)

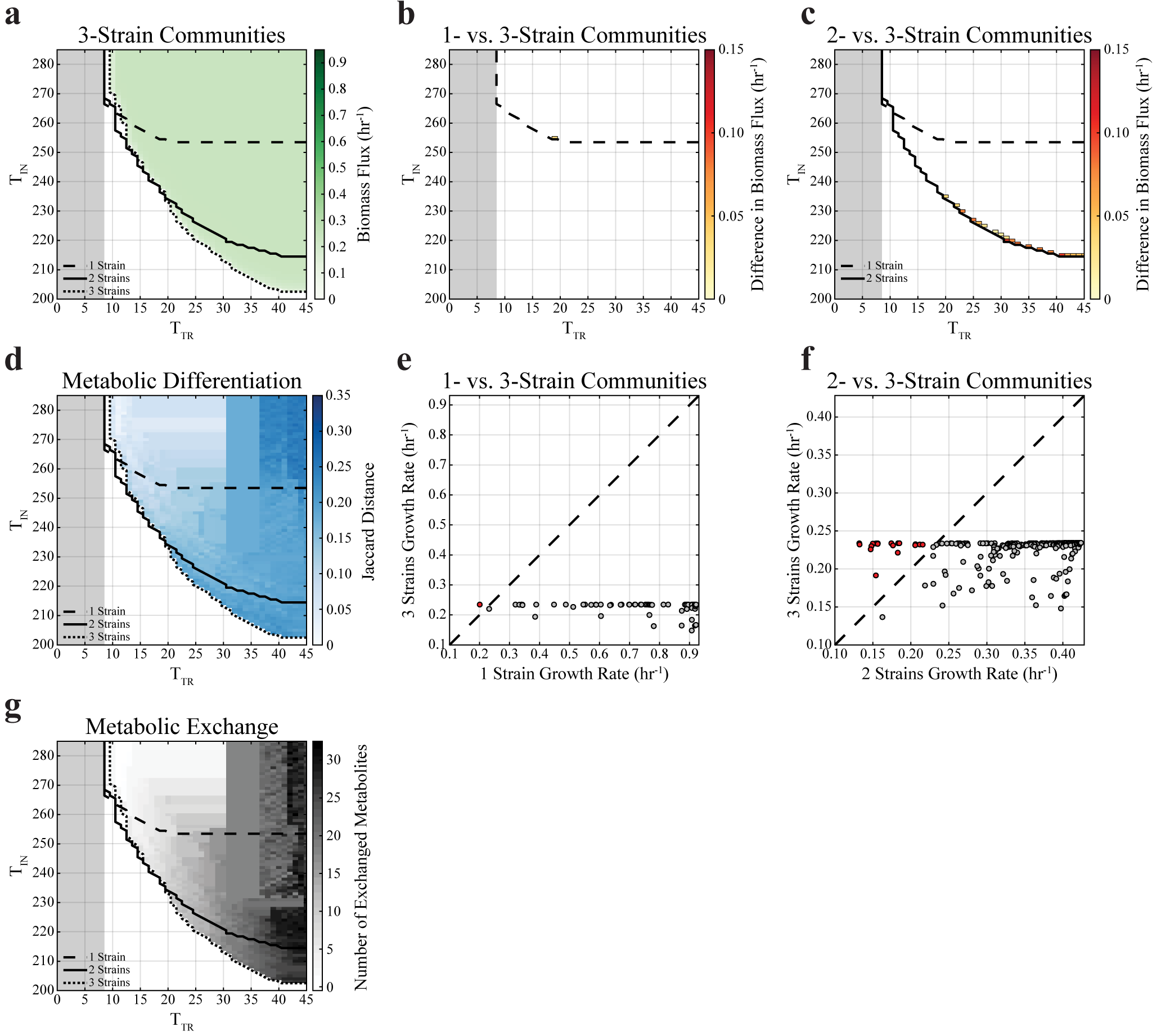

Supplement: FIG S1 [file mSystems.00263-18-sf001.tif]

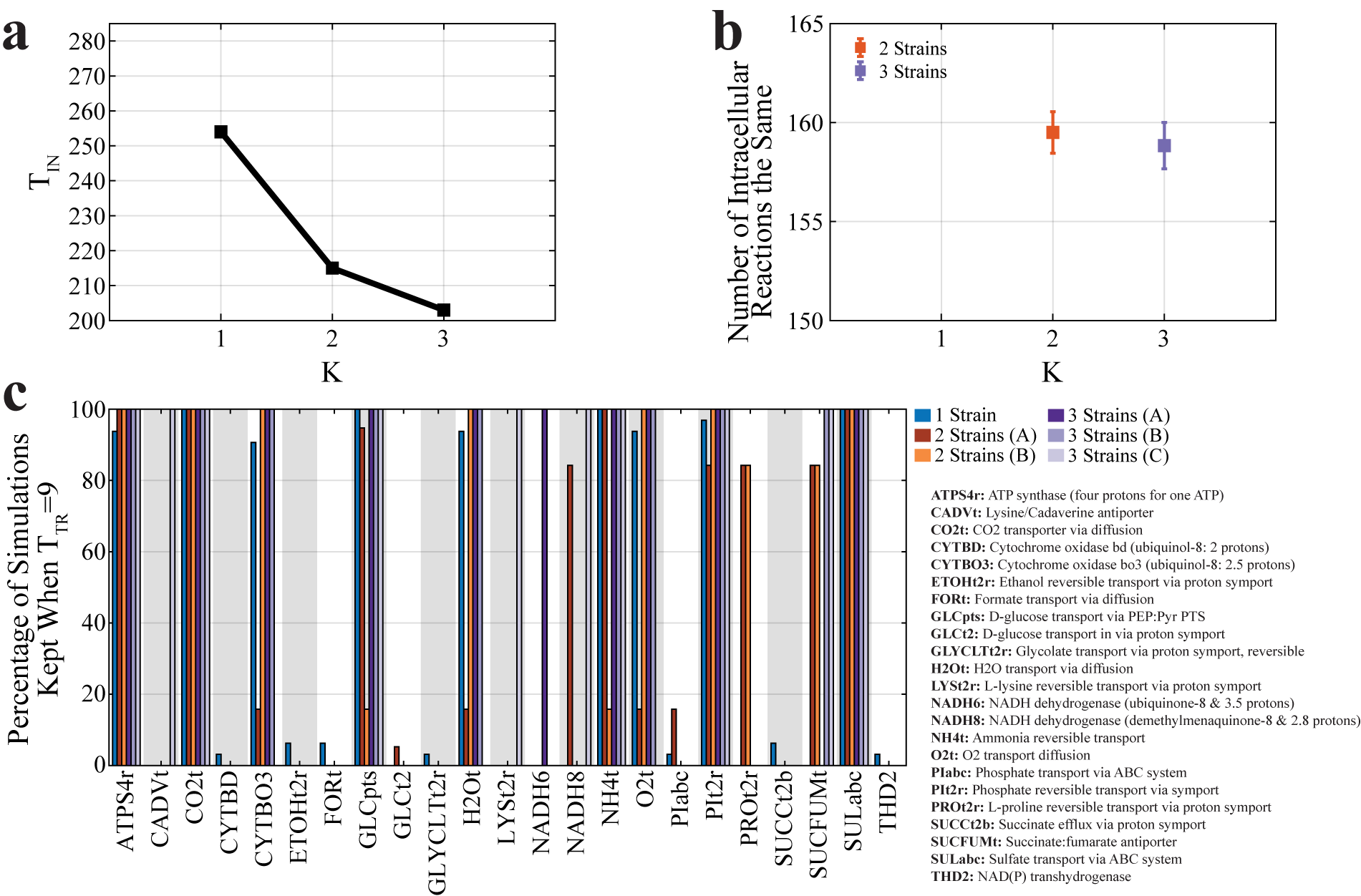

Supplement: FIG S2 [file mSystems.00263-18-sf002.tif]

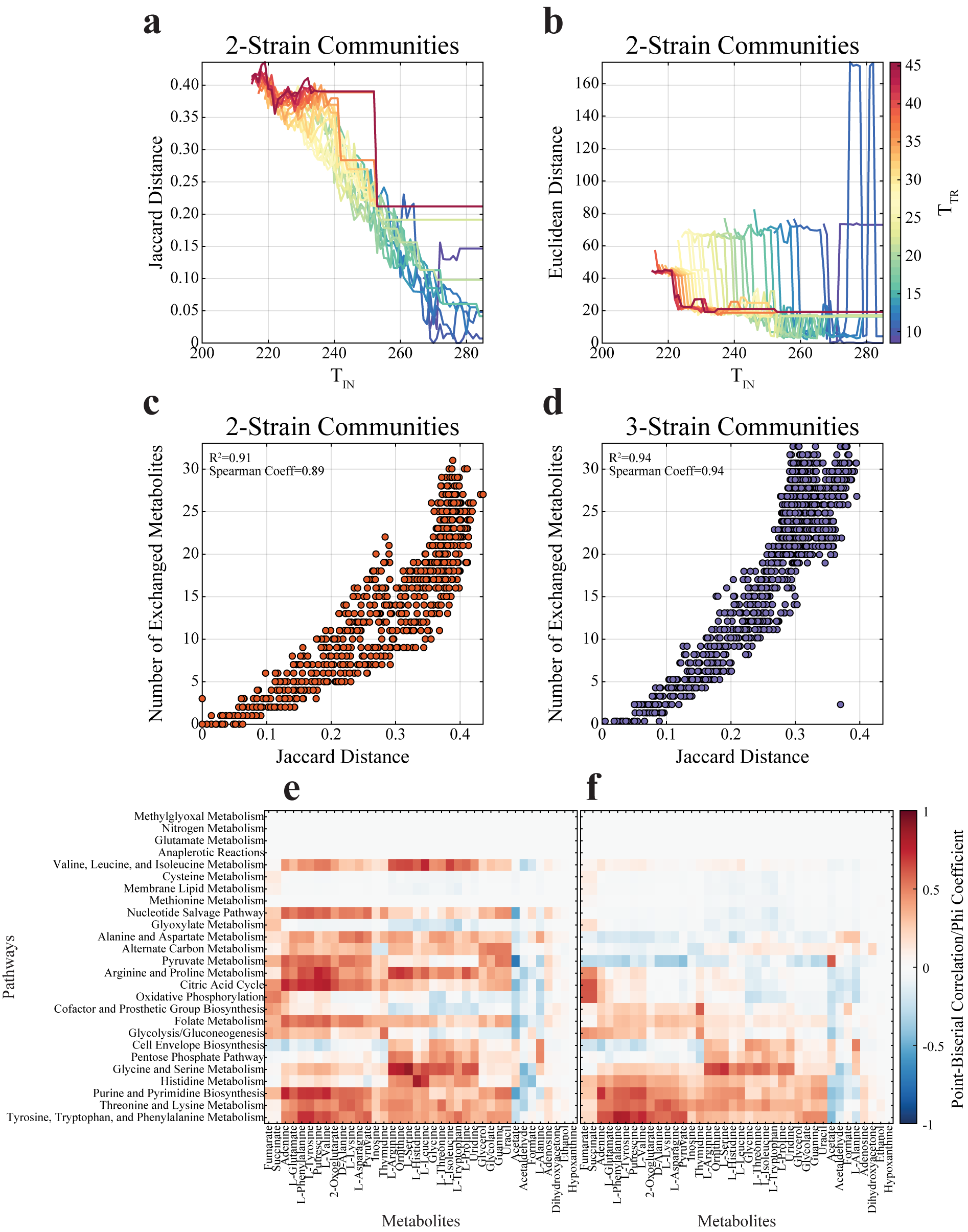

Supplement: FIG S3 [file mSystems.00263-18-sf003.tif]

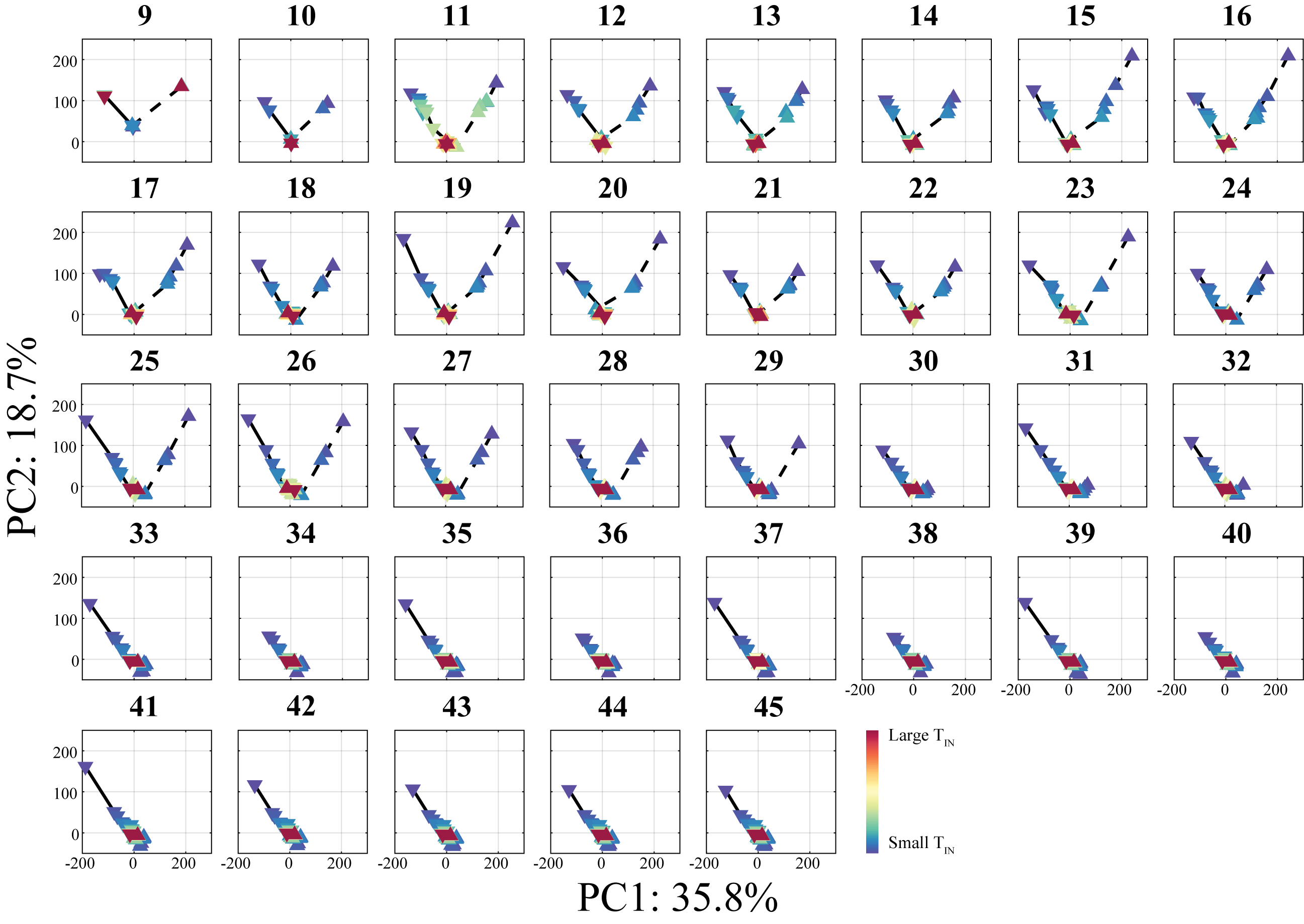

Supplement: FIG S4 [file mSystems.00263-18-sf004.tif]

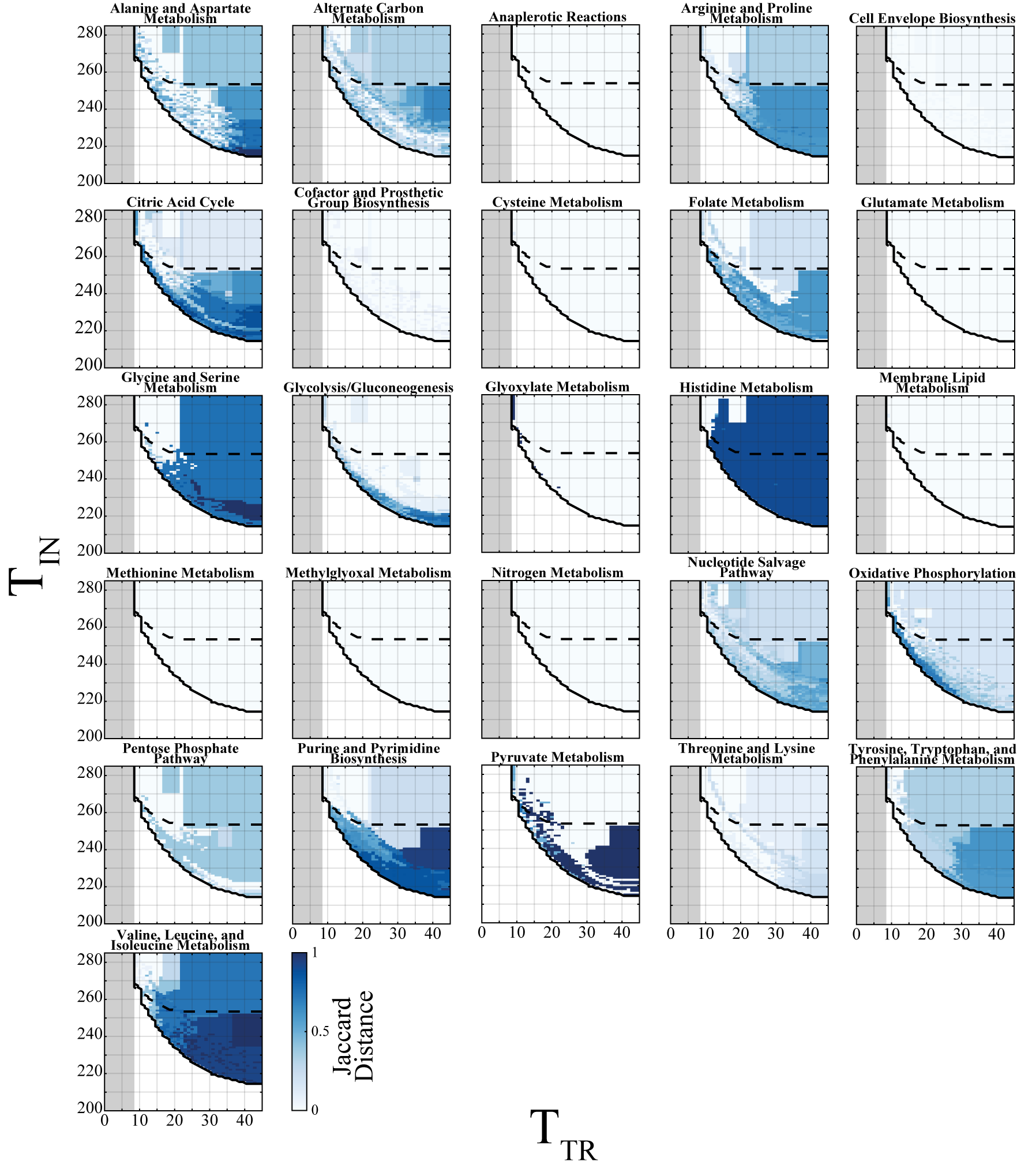

Supplement: FIG S5 [file mSystems.00263-18-sf005.tif]

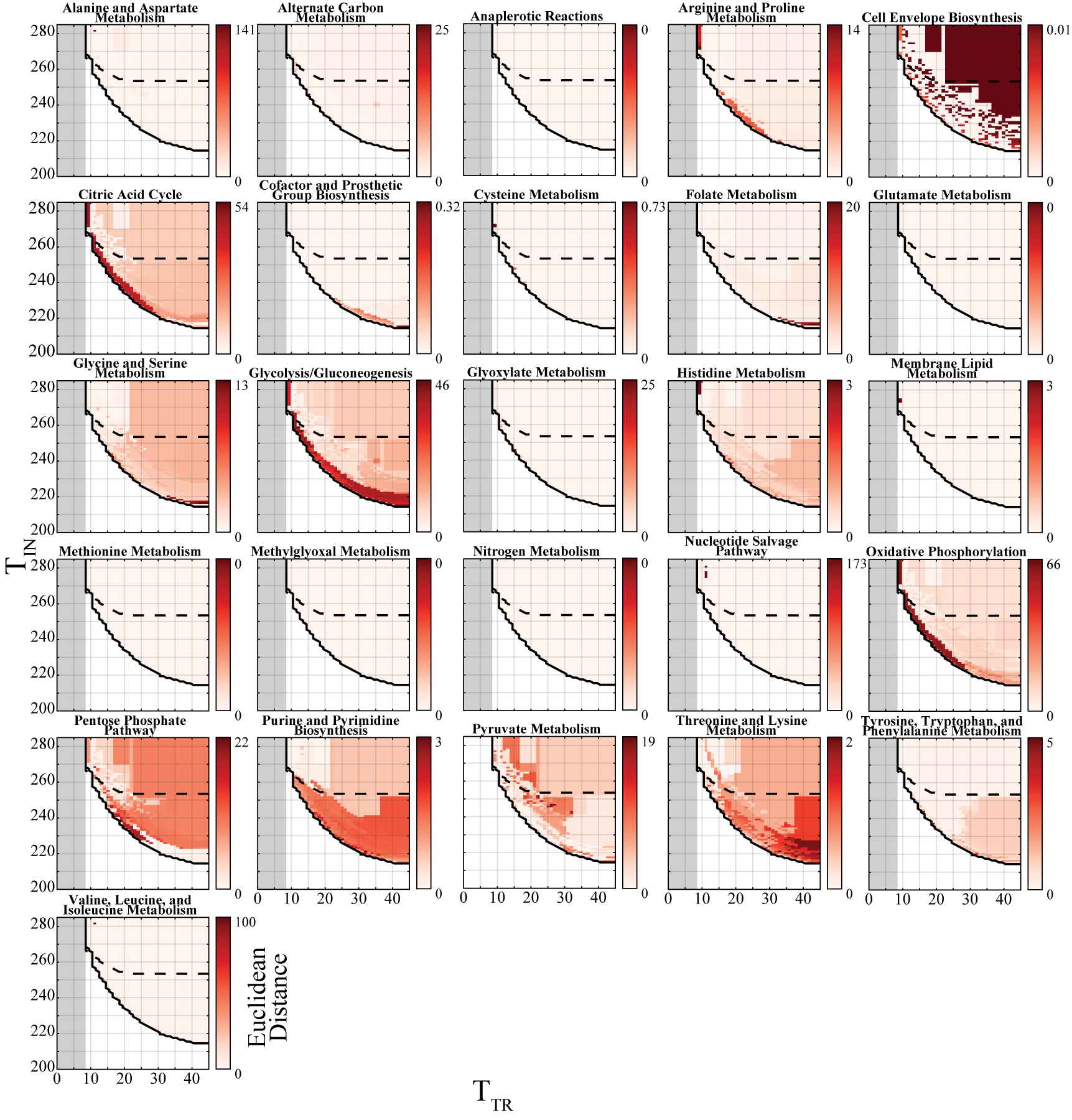

Supplement: FIG S6 [file mSystems.00263-18-sf006.tif]

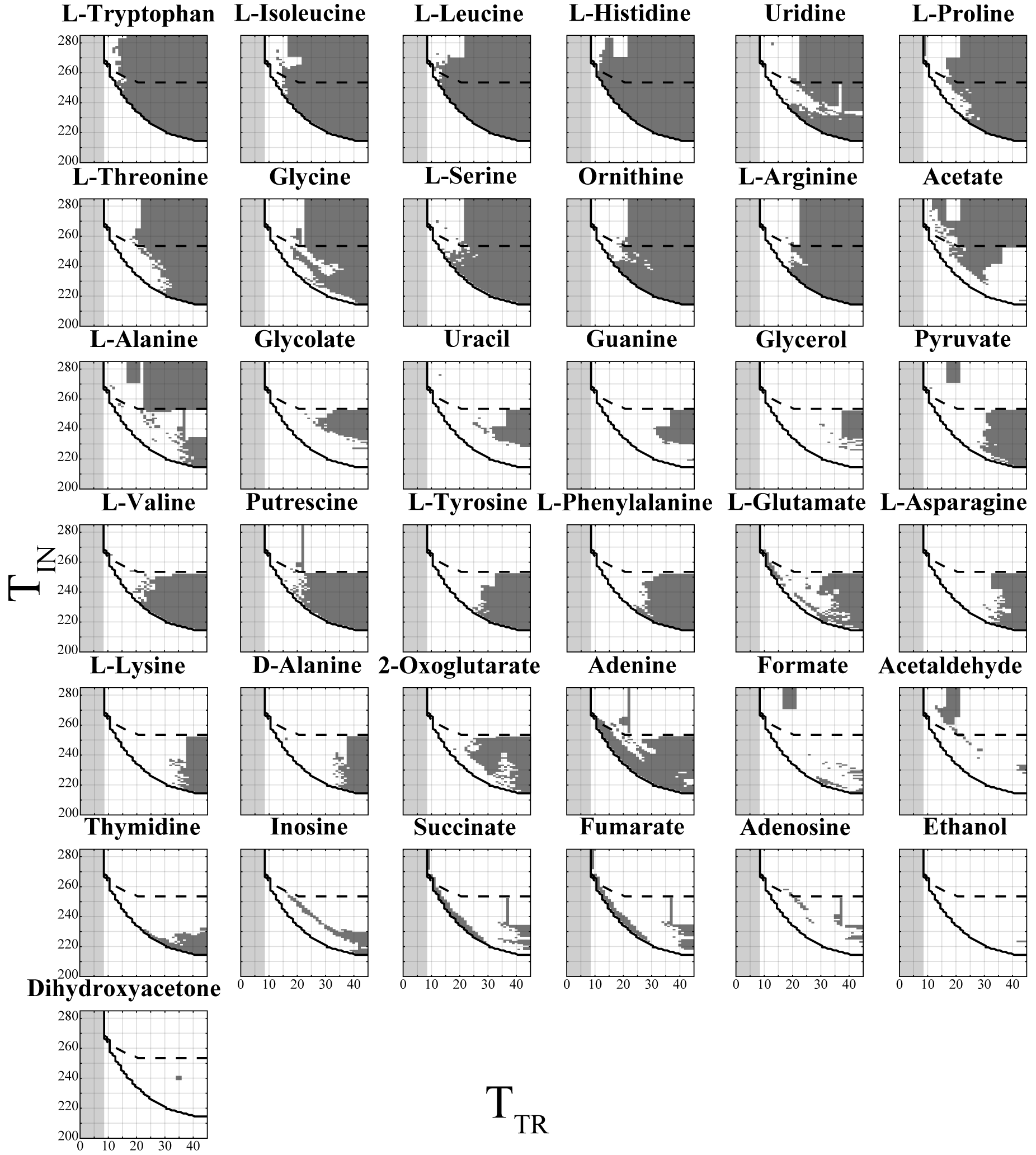

Supplement: FIG S7 [file mSystems.00263-18-sf007.tif]

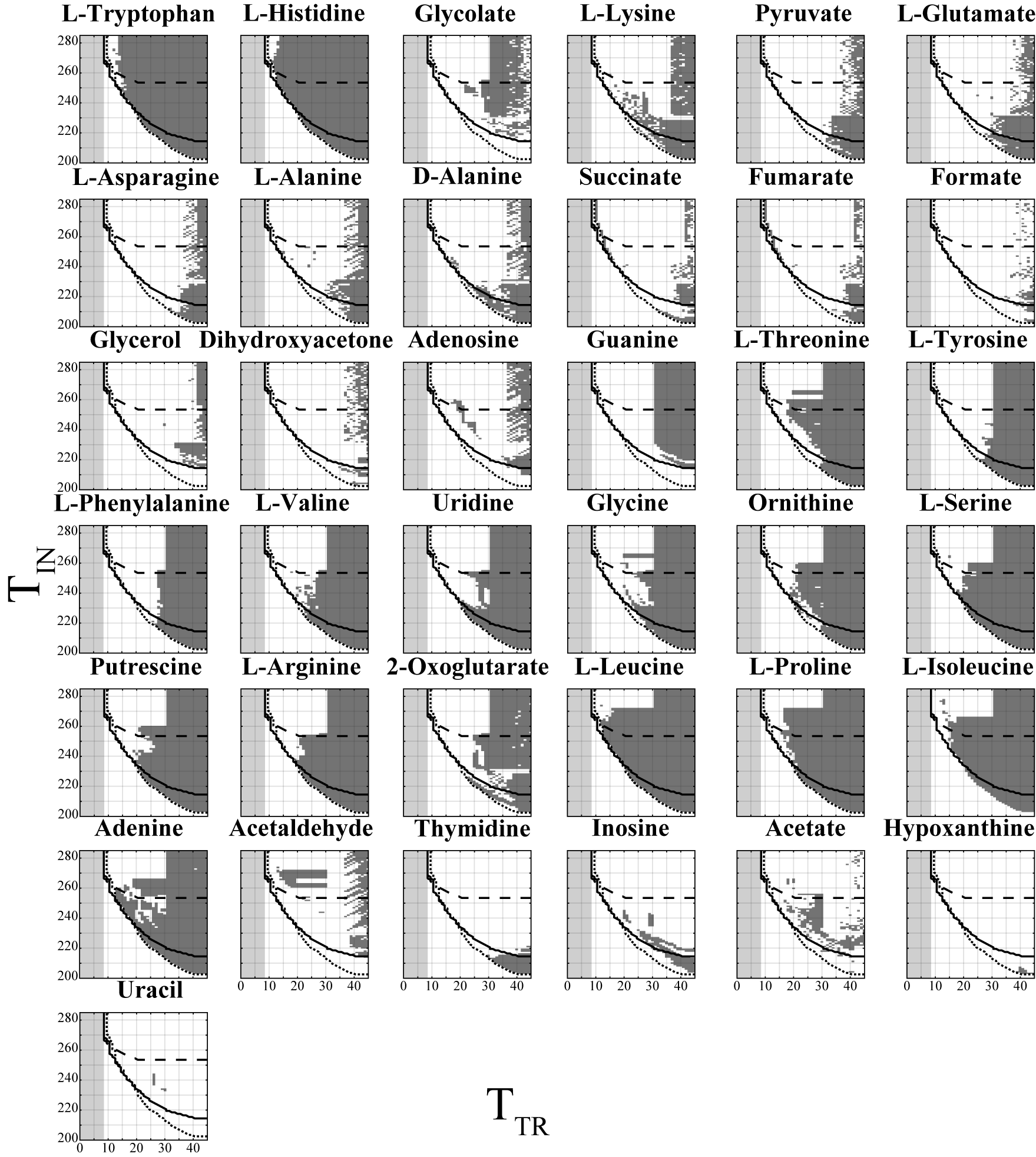

Supplement: FIG S8 [file mSystems.00263-18-sf008.tif]

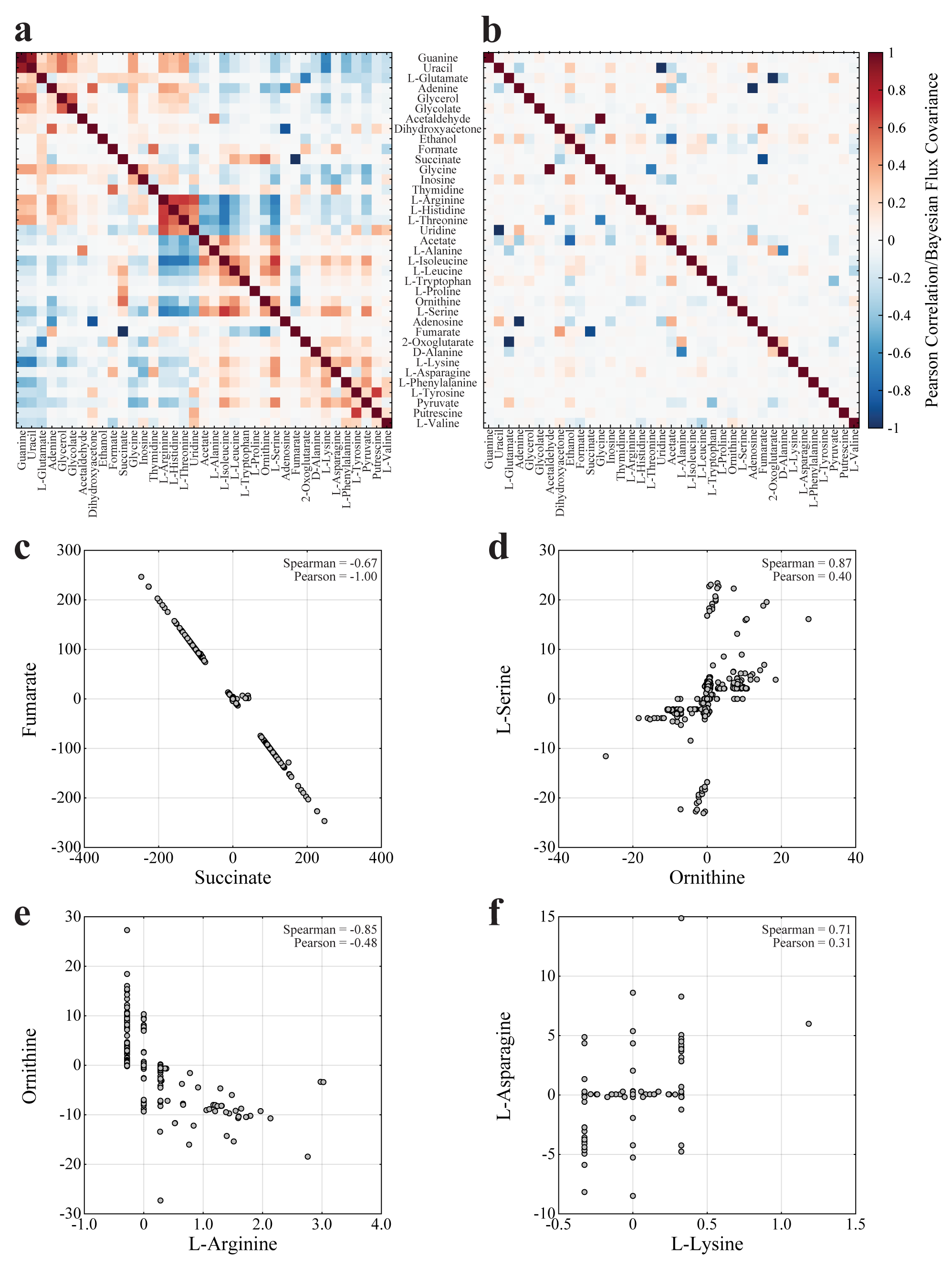

Supplement: FIG S9 [file mSystems.00263-18-sf009.tif]

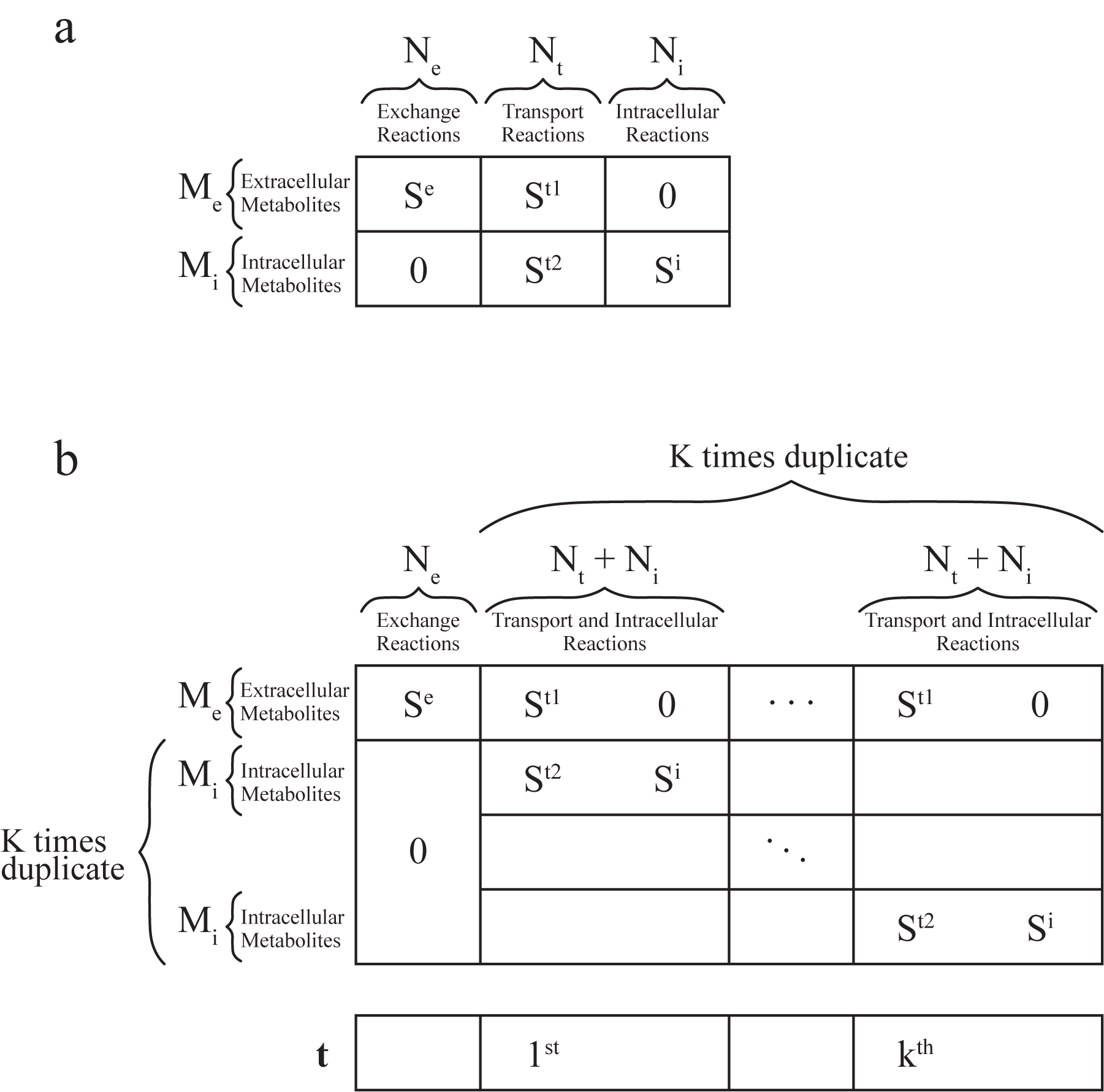

Supplement: FIG S10 [file mSystems.00263-18-sf010.tif]
